# Physiological, Transcriptomic, and Metabolomic Insights into Ammonia-Nitrogen Stress in the Hepatopancreas of *Litopenaeus vannamei* Acclimated to Low Salinity

**DOI:** 10.3390/biology15161394

**Published:** 2026-08-14

**Authors:** Yutong Zhao, Yangyang Ding, Xiaojuan Hu, Qibin Yang, Ziyi Jiang, Yucheng Cao

**Affiliations:** 1 South China Sea Fisheries Research Institute, Chinese Academy of Fishery Sciences, Guangzhou 510300, China; yuton2025@163.com (Y.Z.);; 2 College of Fisheries and Life Sciences, Dalian Ocean University, Dalian 116023, China; 3 Southern Marine Science and Engineering Guangdong Laboratory (Zhuhai), Zhuhai 519082, China; 4 Sanya Tropical Fisheries Research Institute, Sanya 572018, China

**Keywords:** *Litopenaeus vannamei*, low-salinity adaptation, ammonia nitrogen stress, multi-omics analysis, physiology

## Abstract

Elevated ammonia nitrogen significantly reduces the survival rate of *Litopenaeus vannamei* under low-salinity conditions. Low-salinity culture is widely adopted in shrimp farming but susceptible to ammonia accumulation; however, relevant studies mainly focus on juvenile shrimp under single stress or normal salinity. With the aim of exploring how *L. vannamei* responds physiologically and molecularly to elevated ammonia nitrogen after low-salinity adaptation, shrimp were gradually acclimated to 5‰ salinity. They were reared at 5‰ for one week before being exposed to high levels of ammonia nitrogen for 96 h. Under these conditions, the shrimp’s hepatopancreas showed antioxidant imbalance and oxidative damage. Elevated blood ammonia, urea nitrogen and uric acid reflected activated synthesis pathways that clear excess ammonia. Transcriptomic and metabolomic profiling at 12, 48, and 96 h revealed 112 DEGs and pronounced alterations in lipids and amino acid derivatives. Integrated gene-metabolite correlation analysis uncovered three core pathways, along with 11 key DEGs and 17 associated metabolites. In the secretion pathway, solute carrier family 4 (anion exchanger),member 2 (*slc4a3*) was strongly correlated with saquinavir and carnitine. In the metabolism pathway, nicotinamide/nicotinate riboside kinase (*nmrk1*), chitinase (*chia*), Gamma-glutamyltranspeptidase (*ggt1*), UDP-glucose 4-epimerase (*gale*), and spermine oxidase (*smox*) were linked to L-pyroglutamic acid, betaine, etc. In the immune pathway, mitogen-activated protein kinase 8/9/10 (*bsk*) and integrin beta 1 (*cd29*) were associated with leukotriene E4 and niacin. This study reveals that ammonia stress after low-salinity acclimation induces oxidative stress damage, elevated physiological changes in *L. vannamei*, and further elucidates the regulatory pathways underlying its response to ammonia nitrogen stress.

## Simple Summary

Low-salinity farming is popular for *Litopenaeus vannamei* cultivation but easily causes ammonia accumulation that endangers shrimp survival. Most previous studies focus on single stress or normal salinity, while the shrimp response to ammonia stress after low-salinity adaptation remains unclear. Here, we acclimated shrimp to 5‰ salinity and exposed them to high ammonia for 96 h. Ammonia stress caused hepatopancreatic oxidative damage and disturbed hemolymph nitrogen metabolism. Multi-omics analysis revealed 112 differentially expressed genes (DEGs) and altered lipid and amino acid metabolism. Three core secretion, metabolism and immune pathways, together with key gene-metabolite pairs, were identified. This study clarifies the physiological and molecular mechanisms underlying shrimp tolerance to combined low-salinity and ammonia stress, supporting the healthy development of low-salinity aquaculture.

## 1. Introduction

*Litopenaeus vannamei* (*L. vannamei*), a decapod crustacean in the Penaeidae family, originates from Pacific coastal waters across Central and South America [1]. As a globally important cultured shrimp species, it exhibits rapid growth performance and high feed utilization efficiency [2]. Traditional seawater shrimp farming faces challenges of land and water scarcity, along with increasing disease and ecological pressures. Low-salinity estuarine waters are broadly distributed across regions, exhibit consistent water quality, and contain low levels of pathogenic microorganisms [3]. Given *L. vannamei’s* strong adaptability to low-salinity conditions, developing and promoting low-salinity aquaculture can effectively expand farming areas and advance sustainable industry development [3,4]. Common shrimp farming practices in estuarine environments are currently based on three main approaches: intensive recirculating systems, closed zero-water-exchange high-density culture, and ecological earthen pond aquaculture [5,6,7]. Nevertheless, these estuarine aquaculture systems are often hindered by elevated ammonia nitrogen levels and low-salinity stress.

Ammonia is one of the most prevalent pollutants in aquaculture ecosystems. The accumulation of uneaten feed residues and aquatic animal excreta can lead to an elevation of ammonia concentration exceeding safe thresholds, thereby exerting stress on aquatic organisms [8,9]. Notably ammonia nitrogen often reaches 40 mg/L in late-stage intensive shrimp culture [10,11,12]. Furthermore relevant studies on heritability analysis and immune response mechanisms have been carried out under concentrations of no less than 40 mg/L [10,11,12]. High ammonia nitrogen exposure induces histopathological lesions in the crustacean hepatopancreas and gills. Such tissue damage disrupts metabolic homeostasis and increases susceptibility to pathogens. These effects impair growth performance and may ultimately result in mortality [13,14,15]. Existing research on the ammonia nitrogen acclimation capacity of *L. vannamei* pay close attention to biological responses to high ammonia nitrogen. Sustained stress results in higher levels of ammonia, uric acid (UA) and urea nitrogen (UN) in hemolymph [16]. Meanwhile, such stress induces oxidative stress and disrupts basal metabolism (e.g., protein digestion and absorption) and energy homeostasis. Ultimately high ammonia nitrogen stress inhibits growth performance [17,18]. Moreover ammonia nitrogen stress elevates intracellular reactive oxygen species (ROS) levels in hemocytes inducing DNA damage and disrupting DNA repair and autophagy [19]. Such oxidative damage further disrupts the activity balance between antioxidant defense and key enzymes of ammonia metabolism. This can be confirmed by the fluctuating superoxide dismutase (SOD) activity in hepatopancreas and hemolymph [20]; Additionally, the activities of ammonia metabolism-related enzymes in different tissues of *L. vannamei* display a concentration-dependent response to ammonia nitrogen stress. To be specific, the two key enzymes glutamine synthetase (GS) and glutamate dehydrogenase (GDH) in hepatopancreas showed a trend of rising then falling under higher ammonia levels, accompanied by a sharp reduction in transglutaminase (TGase) activity [21]. When the homeostatic balance of ammonia metabolism and detoxification in the hepatopancreas is disrupted, the organism adjusts ammonia excretion through endocrine regulatory pathways. Previous studies have shown that crustacean hyperglycemic hormone (CHH) positively regulates ammonia excretion in *L. vannamei* under ammonia nitrogen stress by activating core ammonia excretion-related genes, including the Rhesus-glycoprotein-type (Rh) ammonia channel and solute carrier family 4 member A (SLC4A) anion exchanger. The inhibition of these excretion-related genes directly leads to ammonia accumulation in the hemolymph and impairs the capacity of ammonia detoxification and excretion [22]. Excessive ammonia accumulation not only disrupts the homeostasis of ammonia metabolism and detoxification but also induces immunosuppression and endoplasmic reticulum (ER) stress injury through multiple signaling pathways. Immunity-related genes, including prophenoloxidase (*proPO*) and integrin β subunit (*ITGB1*), are significantly downregulated [10].

Previous studies have primarily focused on ammonia nitrogen stress under high-salinity conditions (>25‰) or concentrated on small-sized *L. vannamei* (defined as individuals with a body weight of ≤10 g, whereas those with a body weight of >10 g are classified as large-sized shrimp) [17,21,23,24,25]. In summary, the main innovative aspect of this study is the examination of high ammonia-nitrogen stress following low-salinity acclimation. Previous research has primarily focused on single stressors, multi-factor stress (low salinity combined with other environmental stressors), and combined stress, but has not specifically addressed *L. vannamei* following low-salinity acclimation; low-salinity acclimation significantly alters the shrimp’s homeostasis. Furthermore, during the middle and late stages of estuarine aquaculture, larger shrimp exhibit a higher metabolic load and are more sensitive to ammonia-nitrogen accumulation. In our preliminary experiments, we subjected both large and small shrimp to acclimation and stress simultaneously and found that larger shrimp were more sensitive to high-ammonia-nitrogen environments.

In practical low-salinity aquaculture, shrimp that have adapted to local conditions frequently suffer from unexplained sudden mass mortality and muscle clouding [26,27,28]. Without tailored prevention and control protocols in place, such abnormal events have caused considerable financial losses to the industry [26,27,28]. Thus, clarifying the molecular mechanisms underlying tolerance to elevated ammonia nitrogen stress in low-salinity-acclimated *L. vannamei* is of both theoretical and practical importance. In the work, we integrated transcriptomic and metabolomic profiling with enzyme activity measurements and metabolite detection to explore ammonia stress responses in the hepatopancreas of *L. vannamei* acclimated to low salinity. This work clarified the shrimp’s stress-related characteristics and underlying regulatory pathways. The outcomes of this study offer a theoretical framework for refining low-salinity aquaculture practices and advance our understanding of molecular adaptation under ammonia nitrogen stress.

## 2. Materials and Methods

### 2.1. Experimental Shrimp

The experimental *L. vannamei* were collected from the Shenzhen Experimental Base, South China Sea Fisheries Research Institute, CAFS. Before the experiment, all shrimp were acclimated in indoor tanks for 7 days. At the start of the experiment, the shrimp showed consistent feeding activity, with a mean body length of (13.2 ± 0.5) cm and mean body weight of (14.6 ± 0.5) g. During the pre-culture period, water quality parameters were stably maintained at the following levels: temperature (30 ± 0.2) °C, pH 7.2–7.3, salinity (27 ± 1)‰, dissolved oxygen (DO) concentration ≥ 19.24 mg/L, ammonia nitrogen concentration ≤ 0.03 mg/L, and nitrite nitrogen concentration ≤ 0.08 mg/L.

### 2.2. Low Salinity Acclimation Process

Prior to exposure to ammonia nitrogen stress, the shrimp were acclimated to the experimental low-salinity environment (5‰) for 7 days. During this period, the salinity was gradually reduced from 25‰ to 5‰ within 7 days, decreasing by 5‰ daily for the first 2 days and by 2‰ daily for the subsequent 5 days, followed by 7 days of stable acclimation at 5‰. This acclimation procedure was adopted according to previous reports [29,30], which confirmed that a 7-day low-salinity adaptation period enables *L. vannamei* to complete osmoregulatory physiological adjustment without chronic stress. Mortality of the shrimp remained below 3% throughout the 5‰ acclimation phase, demonstrating that the individuals had successfully acclimated to the low-salinity conditions with no signs of physiological stress.

At the start of the low-salinity phase, the shrimp had an average body length of (14.0 ± 0.5) cm and average body weight of (17.75 ± 0.5) g. Following 7 days of rearing at low salinity, the average body length and weight of the shrimp reached (14.6 ± 0.5) cm and (18.15 ± 0.5) g. The shrimp maintained normal feeding behavior throughout the acclimation period, with no disease or physiological irregularities detected. Major water indicators including pH, ammonia nitrogen, nitrite nitrogen, dissolved oxygen, salinity and water temperature were monitored every day and maintained at levels suitable for the growth of *L. vannamei*.

### 2.3. Acute Ammonia Stress

Ammonia nitrogen stress was simulated by adding ammonium chloride (NH_4_Cl) (Tianjin Damao Chemical Reagent Factory, Tianjin, China) to culture water. A 96-h preliminary acute toxicity assay with nine TAN concentration groups was performed to select the ideal stress level. Mortality statistics and LC_50_ calculation were performed accordingly; the control group mortality was below 3.3%, and the 96 h LC50 value was determined to be 42.32 mg/L via IBM SPSS Statistics 25. This concentration was adopted for the formal stress test, and detailed pre-experimental results are shown in Table S1.

After acclimating to low salinity, intact and healthy shrimp individuals were assigned at random to the ammonia stress group and the blank control group. Based on the determined LC_50_, six replicates were set for the stress group and three replicates for the control group. Every replicate group held 30 individuals in 200 L water. No feed was supplied during the 96-h ammonia stress phase.

All experiments were performed in 500 L cylindrical tanks (diameter 100 cm, height 85 cm) filled with 200 L of 5‰ salinity seawater, which was continuously aerated for 24 h before use. After 48 h of shrimp acclimation, pre-weighed NH_4_Cl was added and fully mixed to establish ammonia stress conditions. The initial TAN concentration was calibrated to (42.3233 ± 0.1) mg/L referring to the national standard method (GB 17378.4-2007). Initial water quality indicators were stably maintained within appropriate ranges. During the 96 h stress period, key water quality parameters were monitored at 0, 12, 48, and 96 h and remained stable, while shrimp mortality was observed every 3 h.

### 2.4. Determination of Ammonia, Urea Nitrogen and Uric Acid in Hemolymph

Surviving *L. vannamei* with normal behaviors after low-salinity acclimation and high ammonia stress were sampled randomly at seven time points (3, 6, 12, 24, 48, 72, 96 h). Three shrimp were collected from the control group at 0 h. Three individuals were collected from the experimental group at every other time point. The 0 h control data served as the baseline for enzyme activity analysis.

Shrimp were briefly anesthetized on ice, and hemolymph was withdrawn using a 1 mL sterile syringe. A 1:1 volume mixture was prepared using hemolymph and self-prepared 3.8% (*w*/*v*) sodium citrate anticoagulant solution. The collected hemolymph was immediately centrifuged at 500× *g* for 10 min at 4 °C, and the supernatant hemolymph was then separated and stored at −80 °C until subsequent analysis. After completing sampling at all time points, all hemolymph samples were promptly sent to Nanjing Jiancheng Bioengineering Institute for batch determination of biochemical indices. All experimental operations were carried out strictly following standard operating procedures. The hemolymph indices detected included malondialdehyde (MDA), glutathione (GSH), blood ammonia, UN, and UA. The corresponding detection wavelengths and units for each index are presented in Table S2.

### 2.5. Analysis of Enzyme Activities in Hepatopancreas

The hepatopancreas tissues of *L. vannamei* were promptly dissected using sterile anatomical instruments immediately after hemolymph collection, and the harvested tissues were rapidly snap-frozen in liquid nitrogen. Referring to previous studies [21,31,32], five enzyme activities in the hepatopancreas were determined in this study, including antioxidant-related enzymes (Catalase (CAT); SOD), ammonia metabolism-related enzymes (GDH; GS), and a detoxification-related enzyme (γ-Glutamyltransferase (γ-GT)). Upon completion of sampling at all time points, all tissue samples were immediately sent to Nanjing Jiancheng Bioengineering Institute for batch detection of enzyme activities. All experimental steps were carried out strictly following the manufacturer’s official guidelines. All tissues were homogenized on ice at a 1:10 (g:mL) ratio with pre-cooled normal saline (GS used dedicated extraction solution). SOD, CAT and γ-GT samples were centrifuged at 2500 rpm for 10 min, GDH at 8000× *g* (4 °C, 10 min), and GS at 5000× *g* (25 °C, 10 min). Reaction incubations were conducted at 37 °C for SOD (40 min), CAT (10 min) and γ-GT, and 25 °C for GS (30 min) and GDH. All samples were assayed individually without pooling. The detection wavelength and unit for each enzyme are presented in Table S2. Enzyme activity data and metabolite contents were expressed as mean ± SD (error bars represent SD), with statistical analysis via GraphPad Prism 10.1.2.

### 2.6. Transcriptomic Sampling and Analysis

For the ammonia nitrogen stress experiment, sampling of surviving individuals was carried out at 12, 48, and 96 h after exposure. At each time point, dead or visibly abnormal shrimp were excluded, and six healthy individuals were randomly selected as biological replicates. Prior to dissection, shrimp were anesthetized briefly at low temperature, and hepatopancreas tissues were harvested according to standard protocols. Collected tissues were preserved by snap-freezing in liquid nitrogen right away. The resulting hepatopancreas samples (80–100 mg each) were sent to Guangzhou OZ Biotechnology Co., Ltd. (Guangzhou, China) for transcriptome sequencing analysis.

RNA samples were tested for integrity, purity and concentration by agarose gel electrophoresis, NanoPhotometer (NanoDrop 2000, Thermo Fisher Scientific, Waltham, MA, USA) and Agilent 2100 Bioanalyzer (Agilent Technologies, Santa Clara, CA, USA) prior to transcriptome analysis. Samples possessing a RIN of no less than 7.0 were adopted for library building and sequencing. Library construction included mRNA enrichment, RNA fragmentation and cDNA synthesis. Raw reads were generated via paired-end sequencing using the Illumina NovaSeq X Plus platform with a read length of 150 bp (PE150). Following the removal of low-quality data and adapter sequences via quality control filtering, high-quality clean reads were retrieved. The clean reads were mapped to the *L. vannamei* reference genome (GCF_042767895.1_ASM4276789v1, https://www.ncbi.nlm.nih.gov/assembly/GCF_042767895.1/, accessed on 11 August 2026) downloaded from NCBI. We assessed inter-sample correlation and intra-group repeatability to verify the credibility of biological replicates.

Transcriptome data processing steps included quality filtering of raw reads with fastp v0.23.2, removal of ribosomal RNA sequences using Bowtie2 v2.4.5, and alignment of clean reads to the reference genome with HISAT2 v2.2.1. StringTie v2.2.1 was used for transcript assembly, and RSEM v1.3.3 for gene quantification. Differential gene expression analysis was carried out in the R v4.2.1 environment with the DESeq2 and edgeR packages: DESeq2 v1.36.0 (for datasets with biological replicates) and edgeR v3.38.4 (for datasets without biological replicates). Genes satisfying |log_2_FC| > 1 and FDR < 0.05 were identified as DEGs. Subsequent enrichment analyses were conducted on these genes for GO functional terms (http://geneontology.org/) and KEGG metabolic pathways (https://www.genome.jp/kegg/, accessed on 11 August 2026). Additional R packages for visualization included pheatmap v1.0.12, gmodels v2.18.1, and ropls v1.30.0.

### 2.7. Metabolomic Sampling and Analysis

The sampling method and sample weight were consistent with those used for transcriptomic analysis. Collected hepatopancreas samples (80–100 mg per sample) were delivered to Guangzhou OZ Biotechnology Co., Ltd. for untargeted metabolomics analysis based on liquid chromatography-mass spectrometry (LC-MS).

Analyses employed an Agilent 1290 Infinity LC (Agilent Technologies, Santa Clara, CA, USA) coupled to AB Sciex Triple TOF 6600 (AB Sciex LLC, Framingham, MA, USA) mass spectrometer, using ACQUITY UPLC BEH Amide column (2.1 mm × 100 mm, 1.7 μm) (Waters Corporation, Milford, MA, USA). ESI dual-mode parameters: Gas1 60 psi, Gas2 60 psi, CUR 30 psi, source temperature 600 °C, ISVF ± 5500 V. Metabolites were annotated by accurate precursor mass (<10 ppm) and experimental MS/MS-fragment matching against an in-house database; CAMERA annotated isotopes/adducts to filter false signals. Raw data were converted with ProteoWizard MSConvert; peak detection and grouping were performed using XCMS v3.20.0 and CAMERA v1.52.0. Multivariate and enrichment analyses were conducted in R v4.2.1 (R Foundation for Statistical Computing, Vienna, Austria) with three packages: gmodels (v2.18.1), ropls (v1.30.0) and pheatmap (v1.0.12). PCA, PLS-DA and OPLS-DA revealed intergroup metabolic divergence, and Pearson correlation analysis evaluated the reproducibility of biological replicates. Hierarchical clustering was performed via the pheatmap package. Metabolites with OPLS-DA VIP ≥ 1 and Student’s *t*-test *p* < 0.05 were regarded as significantly differential compounds. GO and KEGG enrichment analyses were performed by hypergeometric tests with Q-value ≤ 0.05 as the significance threshold.

### 2.8. Integrated Transcriptomic and Metabolomic Analysis

Finally a target pathway-centered multi-omics integration strategy was adopted. Pearson correlation analysis was applied to assess the relationships between DEGs and differentially accumulated metabolites (DAMs). Correlation network subgraphs were constructed. Based on preliminary screening of DEGs and DAMs a threshold of |r| ≥ 0.5 and *p* < 0.05 was set. This threshold identified key DEGs and DAMs involved in target pathways. The correlations and core roles of these key DEGs and DAMs were further displayed and summarized in a table.

### 2.9. Quantitative Real-Time PCR

To obtain stable and reliable data, hepatopancreas samples were collected from six shrimps in the control group and ammonia nitrogen stress group at 12 h, 48 h and 96 h post-treatment. At each time point, three tissue samples were randomly selected from both experimental and control groups as biological replicates. Total RNA was extracted separately and subjected to reverse transcription independently. Each cDNA sample was used as the template, and three technical replicates were set for each sample for qRT-PCR verification. The qRT-PCR amplification program was as follows: initial denaturation at 95 °C for 3 min; 40 cycles of 95 °C for 10 sec, annealing at 58 °C for 30 sec, and extension at 72 °C for 20 sec. Melting curve analysis was performed from 60 °C to 95 °C after amplification. According to previous references, *β-actin* was selected as the reference gene. In this study, 12 differentially expressed genes randomly screened from five key pathways were validated via qRT-PCR.

Quantitative real-time PCR (qPCR) amplification was implemented on a CG-02 real-time fluorescence PCR system (Heal Force, China) using SYBR Green Master Mix (Accurate Biology, Changsha, Hunan, China). Samples were measured in three technical replicates. Primer sequences are available in Table S3, which were designed using NCBI Primer-BLAST (https://www.ncbi.nlm.nih.gov/tools/primer-blast/, accessed on 11 August 2026). Melting curve analysis verified that all primers amplified target genes specifically with single peak signals, without non-specific amplification or primer dimers.

All statistical analyses were conducted using two-sided tests. A *p* value < 0.05 was considered statistically significant. In qPCR assays, the 2^−(ΔΔCt)^ method was applied to calculate relative gene expression, and *β-actin* was selected as the reference gene. All statistical computations and graph generation were completed via Microsoft Excel 2021 and GraphPad Prism 10.1.2. The Shapiro-Wilk test and Levene’s test were used to assess data normality and homogeneity of variance, respectively. All *p* values were greater than 0.05. On this basis, two-tailed *t*-tests were used to compare fold changes of qRT-PCR and RNA-seq data.

## 3. Results

### 3.1. Hemolymph Biochemical Changes Under Ammonia Nitrogen Stress

**Figure 1:**
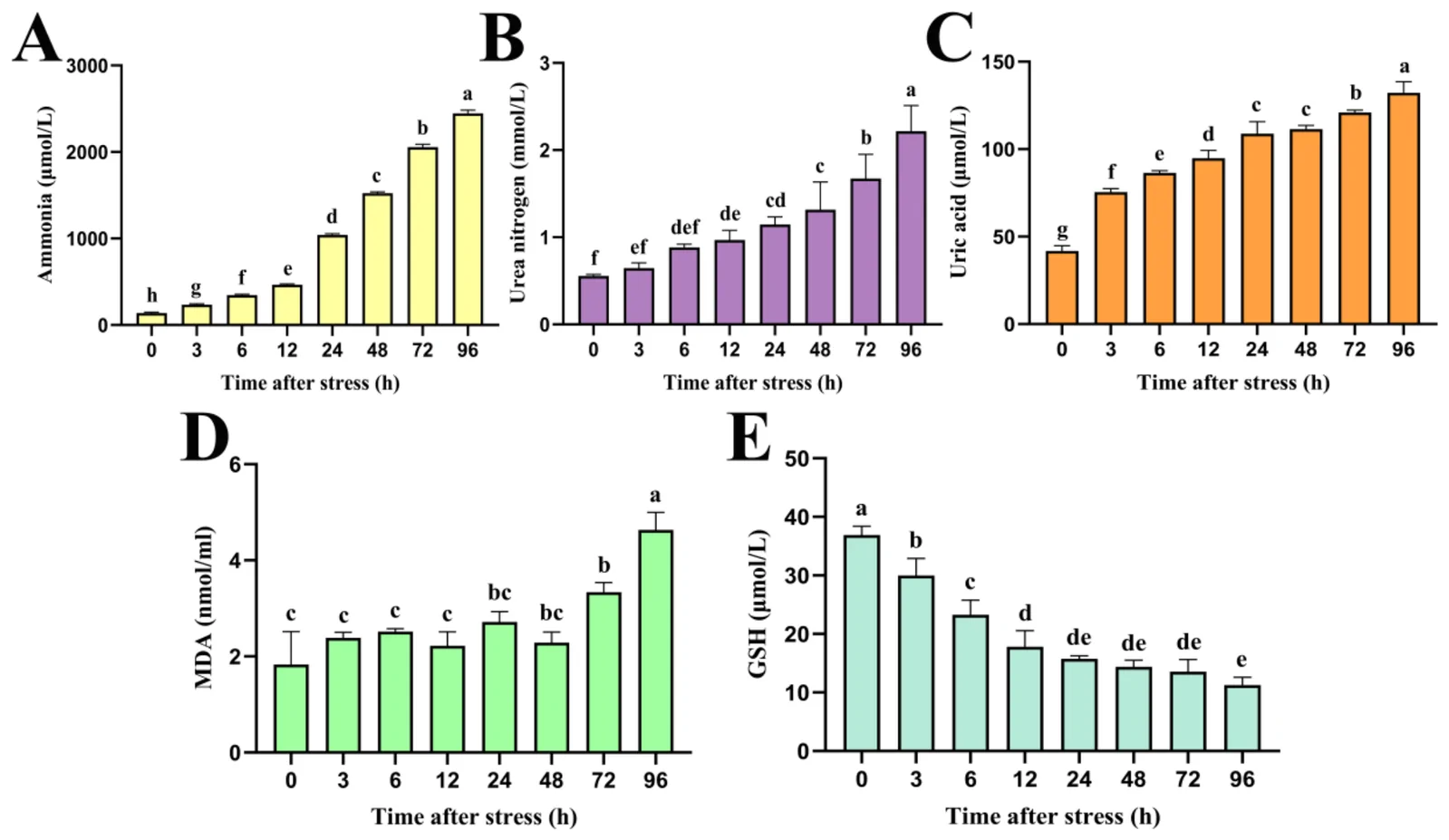
Metabolic products in the hemolymph of *L. vannamei* following 96 h of acute ammonia stress after low-salt acclimation. (**A**) This bar chart shows the ammonia concentration in the hemolymph of *L. vannamei*. (**B**) This bar chart shows the urea nitrogen concentration in the hemolymph of *L. vannamei*. (**C**) This bar chart shows the uric acid concentration in the hemolymph of *L. vannamei*. (**D**) This bar chart shows the MDA content in the hemolymph of *L. vannamei*. (**E**) This bar chart shows the GSH concentration in the hemolymph of *L. vannamei*. Each bar represents the mean ± standard error of the mean (SEM) (n = 3). Data groups labeled with different letters differ significantly (*p* < 0.05).

To assess overall responses of *L. vannamei* to high ammonia nitrogen stress after low-salinity acclimation, shrimp stress status at each time point was evaluated by measuring plasma biochemical component contents. Regular variations were observed in plasma nitrogen metabolites and oxidative stress-related indices of low-salinity-acclimated *L. vannamei* during high ammonia nitrogen stress (Figure 1). With prolonged stress duration, hemolymph ammonia, urea nitrogen, MDA, and uric acid contents increased gradually, while GSH content decreased continuously.

Specifically, hemolymph ammonia concentration increased markedly post-exposure and peaked at 96 h of stress (Figure 1A). Urea nitrogen content also showed a time-dependent elevation, being significantly higher than the initial level at 12 h post-stress (*p* < 0.05), with a more pronounced increase in the late stress stage (Figure 1B). Hemolymph uric acid levels rose gradually with stress progression and increased significantly from 3 h post-stress (*p* < 0.05), indicating accumulation of nitrogen metabolism end products under ammonia nitrogen stress (Figure 1C).

For oxidative stress indices, MDA content rose progressively over the stress period and was markedly elevated relative to baseline at 24 h and 72 h (*p* < 0.05), indicating continuous aggravation of lipid peroxidation (Figure 1D). In contrast, hemolymph GSH content declined continuously over the stress period, dropping significantly below the 0 h control from 3 h onward (*p* < 0.05) and remaining low thereafter (Figure 1E).

### 3.2. Changes of Enzyme Activities in Hepatopancreas

To explore high ammonia nitrogen stress effects on the hepatopancreas of low-salinity acclimated *L. vannamei*, we determined the dynamic changes in the activities of antioxidant, ammonia metabolism-related, and detoxification-related enzymes at different stress time points to visually characterize the physiological responses of shrimp to ammonia nitrogen exposure.

**Figure 2:**
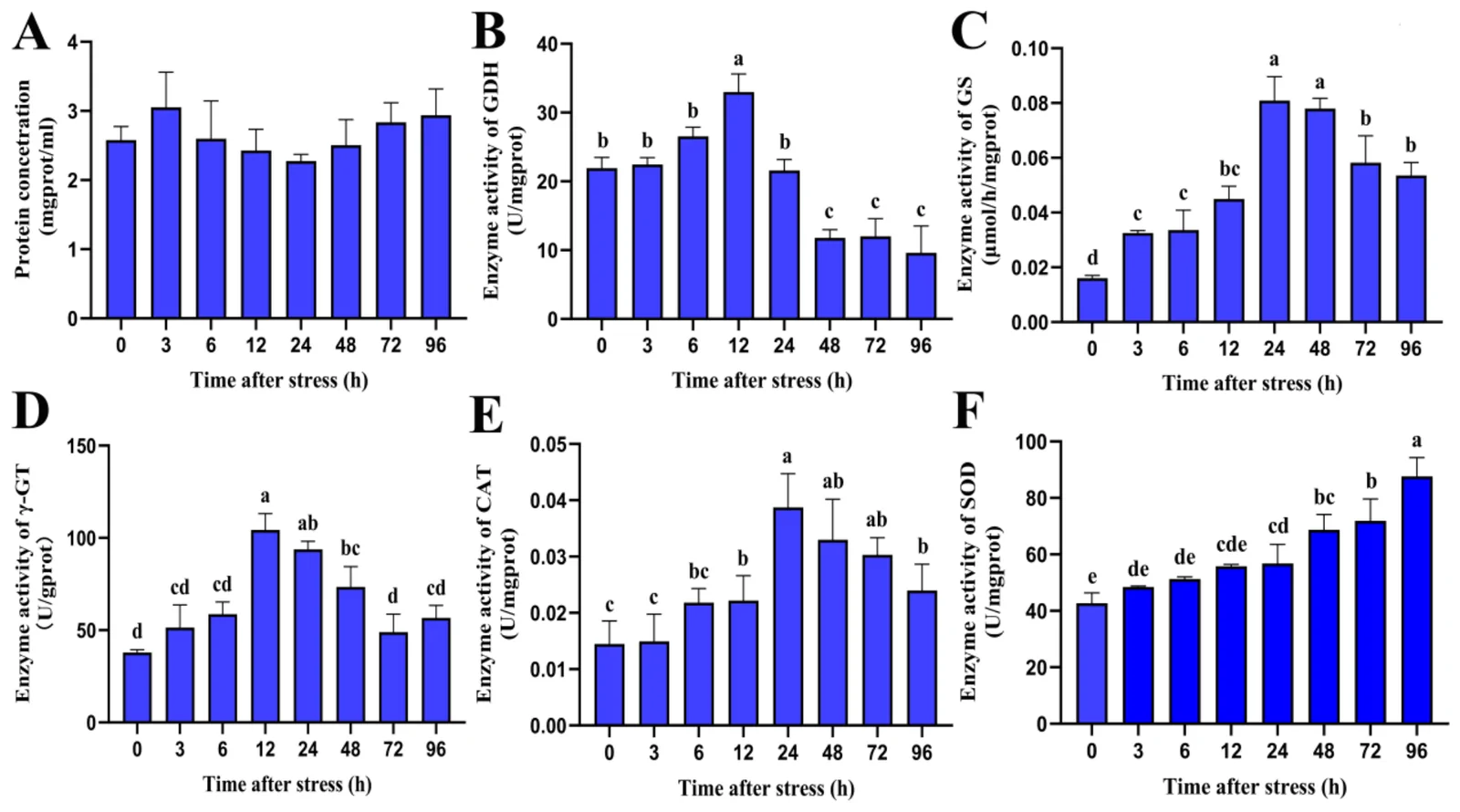
Physiological enzyme activities in the hepatopancreas of *L. vannamei* under low-salinity acclimation and high ammonia nitrogen stress over 96 h. (**A**) Hepatopancreatic protein concentration; (**B**) GDH activity; (**C**) GS activity; (**D**) γ-GT activity; (**E**) CAT activity; (**F**) SOD activity. All data are presented as mean ± standard error of the mean (mean ± SEM), with three biological replicates (n = 3). Values marked with different lowercase letters are significantly different at *p* < 0.05.

Figure 2A depicts the protein content in the hepatopancreas of shrimp subjected to low-salinity acclimation and successive high ammonia nitrogen stress. Among ammonia metabolism-related enzymes, GDH activity exhibited an overall trend of initial increase followed by decrease, peaking significantly at 12 h (*p* < 0.05) (Figure 2B). GS activity showed a similar rise-then-fall pattern, with a significant peak at 24 h of stress (Figure 2C).

For detoxification-related enzymes, γ-GT activity increased first and then declined, reaching a significant maximum at 12 h (*p* < 0.05) followed by a gradual decrease (Figure 2D). Regarding antioxidant enzymes, CAT activity initially rose and then dropped, reaching the highest level at 24 h (*p* < 0.05) and remaining higher than the control group (Figure 2E). In contrast, SOD activity showed a continuous upward trend, increasing significantly from 24 h onward (*p* < 0.05) (Figure 2F).

### 3.3. Quality Evaluation of Transcriptome Sequencing

Transcriptome sequencing was conducted on 36 hepatopancreas samples to investigate gene expression alterations in low-salinity-adapted *L. vannamei* exposed to high ammonia nitrogen stress. In total, 165 GB of high-quality clean data were produced. All samples were divided into six groups, including three control groups (12 h-CK, 48 h-CK, 96 h-CK) and three stress treatment groups (12 h, 48 h, 96 h), with 6 biological replicates per group. Sequencing quality evaluation, including raw data, clean data and GC content, indicated consistent and high-quality sequencing results with good reproducibility among replicates (Table 1 and Table S4).

Furthermore, PCA based on Pearson correlation (Figure S1A) and correlation heatmap (Figure S1B) showed clear clustering of samples within each group. The results also demonstrated high reproducibility and strong correlations of replicates within each group. Conversely, prominent disparities and clear segregation appeared between the two groups. Collectively, These results verify biological repeat validity and sequencing data reliability.

### 3.4. Profiling of Differentially Expressed Genes

TPM normalization and volcano plots were used to compare hepatopancreatic gene expression at 12 h, 48 h and 96 h across control and high ammonia nitrogen groups. Ultimately, 24,066 annotated genes were matched against the reference genome. A total of 25,305 genes, including 1239 novel genes, were detected in the hepatopancreas samples, and 25,320 genes were identified as expressed genes according to TPM quantification results (Figure S2).

**Figure 3:**
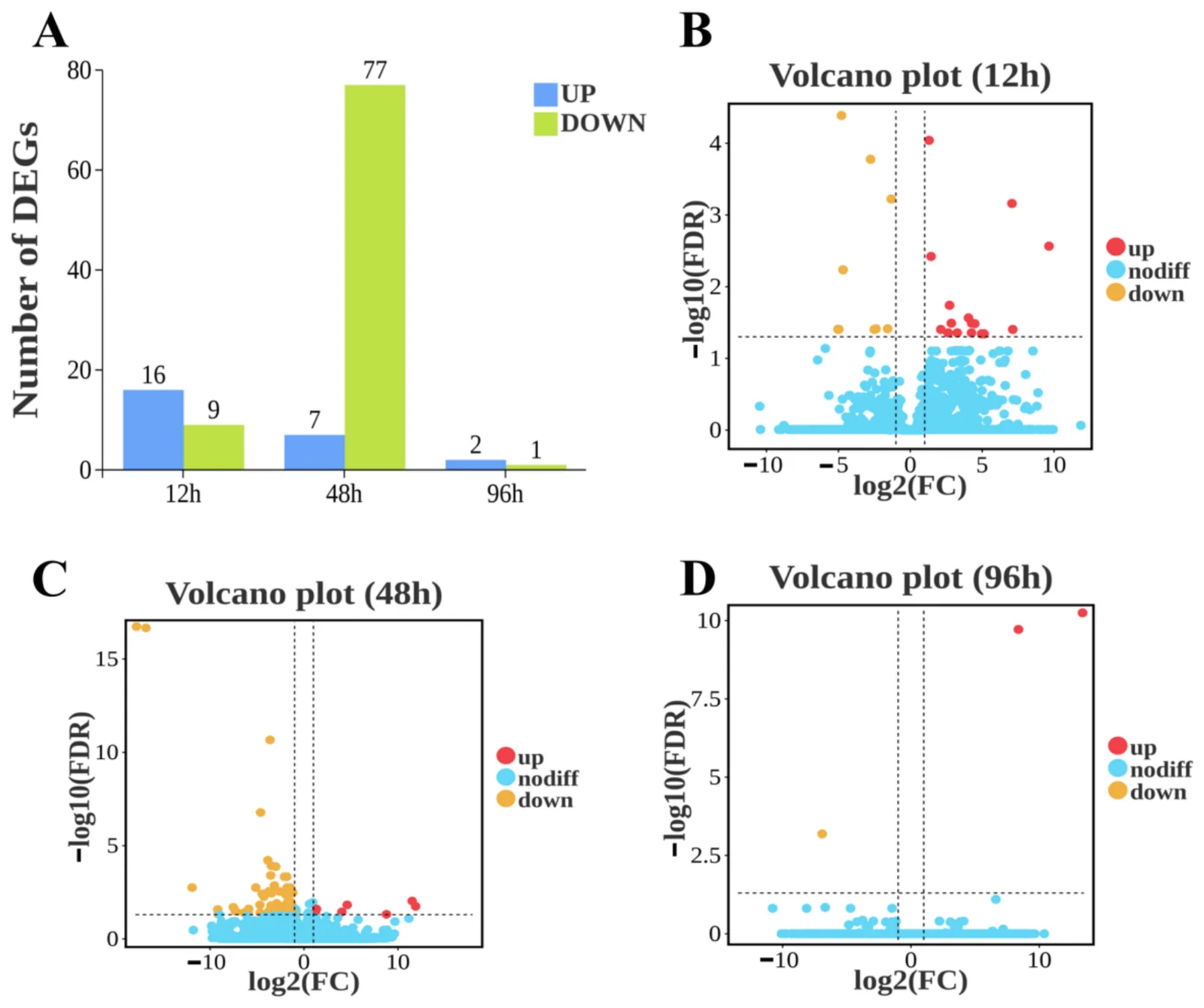
Overview of DEGs. (**A**) Quantities of up- and downregulated DEGs at three time points (blue: upregulated; green: downregulated). (**B**–**D**) Volcano plots of DEGs. Significant DEGs were screened by FDR < 0.05 and |log_2_FC| > 1 (The vertical dashed lines represent |log_2_FC| = 1, and the horizontal dashed line represents FDR = 0.05 for screening significant DEGs). Up, down and nodiff represent upregulated, downregulated and non-differentially expressed genes, respectively.

After 12 h, 48 h and 96 h of ammonia stress, 16, 7 and 2 genes were significantly up-regulated, whereas 9, 77 and 1 genes were markedly down-regulated (Figure 3A). Volcano plots further indicated that significant gene expression changes mainly occurred at 12 h and 48 h post stress (Figure 3B–D). Overall, high ammonia stress triggered obvious time-dependent gene expression alterations in low-salinity-acclimated shrimp. Notably, the 48 h stress group presented the strongest transcriptional response with the maximum number of DEGs and an overall downregulated expression trend.

### 3.5. GO Functional Annotation and Enrichment of DEGs

GO functional enrichment analysis was conducted on significant DEGs to identify critical candidate genes and clarify their biological functions.

GO enrichment analysis of DEGs classified these genes into three main categories: cellular component (CC), biological process (BP) and molecular function (MF). Among the main functional groups obtained from the hepatopancreas of *L. vannamei*, cellular process, binding and cellular anatomical entity were the top three subcategories containing the most DEGs at 12 h, 48 h and 96 h after high ammonia nitrogen exposure (Figure S3). Notably, immune-related subcategories (e.g., response to stimulus), metabolism-related subcategory (metabolic process), and protein-related subcategory were significantly enriched in the hepatopancreas. Additionally, low-salinity ammonia nitrogen stress led to prominent enrichment of biological regulation-related functions in hepatopancreas, covering localization, biological regulation, transporter activity and catalytic activity.

### 3.6. KEGG Enrichment Analysis of DEGs

KEGG database annotation of all DEGs was performed to analyze hepatopancreatic pathway responses under combined low-salinity and high ammonia stress. With *p* < 0.05 as the threshold, the top 20 significantly enriched pathways were identified at each sampling time point.

**Figure 4:**
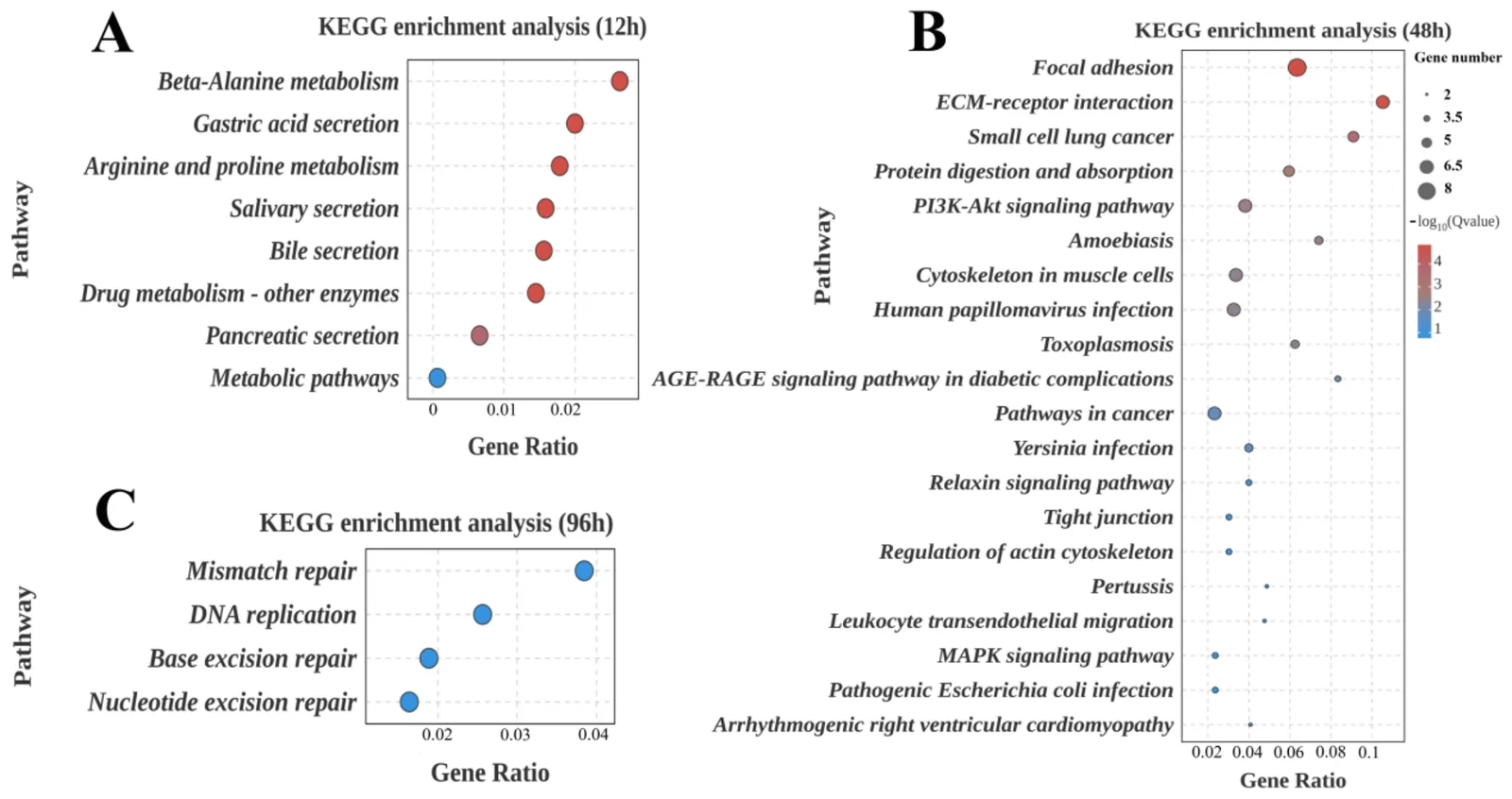
KEGG Pathway Enrichment for Transcriptomics. (**A**–**C**) KEGG enrichment results at 12 h, 48 h and 96 h after combined low-salinity and high ammonia nitrogen stress. Pathways were screened at *p* < 0.05, and the top 20 pathways with the lowest Q-values are shown. The y-axis represents pathway names, and the x-axis indicates enrichment factor. Bubble size reflects the number of enriched DEGs, and darker red denotes higher enrichment significance (lower Q-value).

For *L. vannamei* after low-salinity acclimation, 12-h ammonia nitrogen stress led to prominent enrichment of hepatopancreatic DEGs in many core biological pathways, including multiple metabolic pathways (e.g., β-alanine metabolism, arginine and proline metabolism, and general metabolic pathways) and secretion-related pathways (e.g., gastric acid secretion and pancreatic secretion) (Figure 4A). At 48 h after ammonia nitrogen stress upon low-salinity acclimation, metabolic pathways contained the most DEGs in the hepatopancreas, followed by signaling pathways such as the PI3K-Akt signaling pathway, immune-related pathways (e.g., focal adhesion), and protein digestion and absorption (Figure 4B). After 96 h of stress exposure, transcriptome profiling showed that DEGs from hepatopancreas were largely enriched in damage repair-related pathways, such as mismatch repair and DNA replication (Figure 4C).

Overall, the results revealed five core biological pathways that were significantly enriched in the hepatopancreas of *L. vannamei* under combined low-salinity and high ammonia nitrogen stress: secretion, metabolic pathways, protein digestion and absorption, mismatch repair, and immune-related pathways (Table 1). Furthermore, metabolic pathways harbored the largest number of DEGs in the hepatopancreas across all annotated pathways.

### 3.7. qRT-PCR Validation of Representative DEGs Identified by RNA-Seq

**Figure 5:**
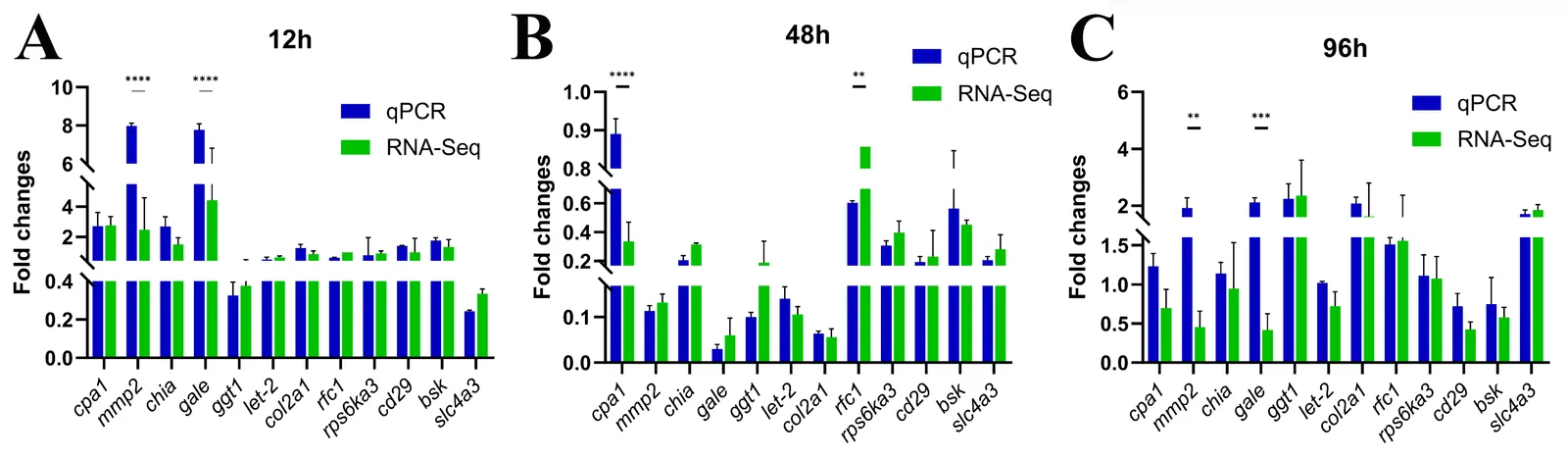
Consistency validation between qRT-PCR and RNA-seq. (**A**–**C**) Fold changes of 12 selected DEGs at 12, 48, and 96 h post-stress, measured by qRT-PCR (blue bars) and RNA-seq (green bars). The x-axis shows validated DEGs, and the y-axis represents expression fold changes relative to the control. Significance levels are annotated as follows: *p* < 0.0001 denoted by “****”; *p* < 0.001 denoted by “***”; *p* < 0.01 denoted by “**”.

To confirm the credibility of transcriptome data, we performed qRT-PCR analysis on hepatopancreas specimens collected from all groups at different stress stages. All gene expression profiles were normalized to the housekeeping gene *β-actin*. The comparable expression fold changes between qRT-PCR and RNA-Seq supported the authenticity of our sequencing data (Figure 5 and Figure S4).

### 3.8. Differentially Accumulated Metabolites

In this work, LC-MS was applied to perform untargeted metabolomics analysis on hepatopancreas samples of *L. vannamei*. Metabolite identification detected 11,802 features in positive ion mode (POS) and 12,375 features in negative ion mode (NEG) (Table S5).

Pearson correlation coefficients were adopted to draw a sample correlation heatmap (Figure S5), so as to assess the reproducibility of metabolomic data. Analysis showed high intra-group correlation coefficients (0.8–0.9) in both POS and NEG modes, while inter-group coefficients ranged from 0.5 to 0.6. The above results prove that the metabolomic data have good reproducibility, and distinct metabolic differences exist between the experimental group and the control group.

### 3.9. Quantitative Analysis of Differentially Accumulated Metabolites

To characterize differential metabolites in the hepatopancreas of *L. vannamei* under low-salinity acclimation and 42.32 mg/L ammonia nitrogen stress, we performed pairwise comparisons between stress and control groups at 12, 48 and 96 h post-treatment.

**Figure 6:**
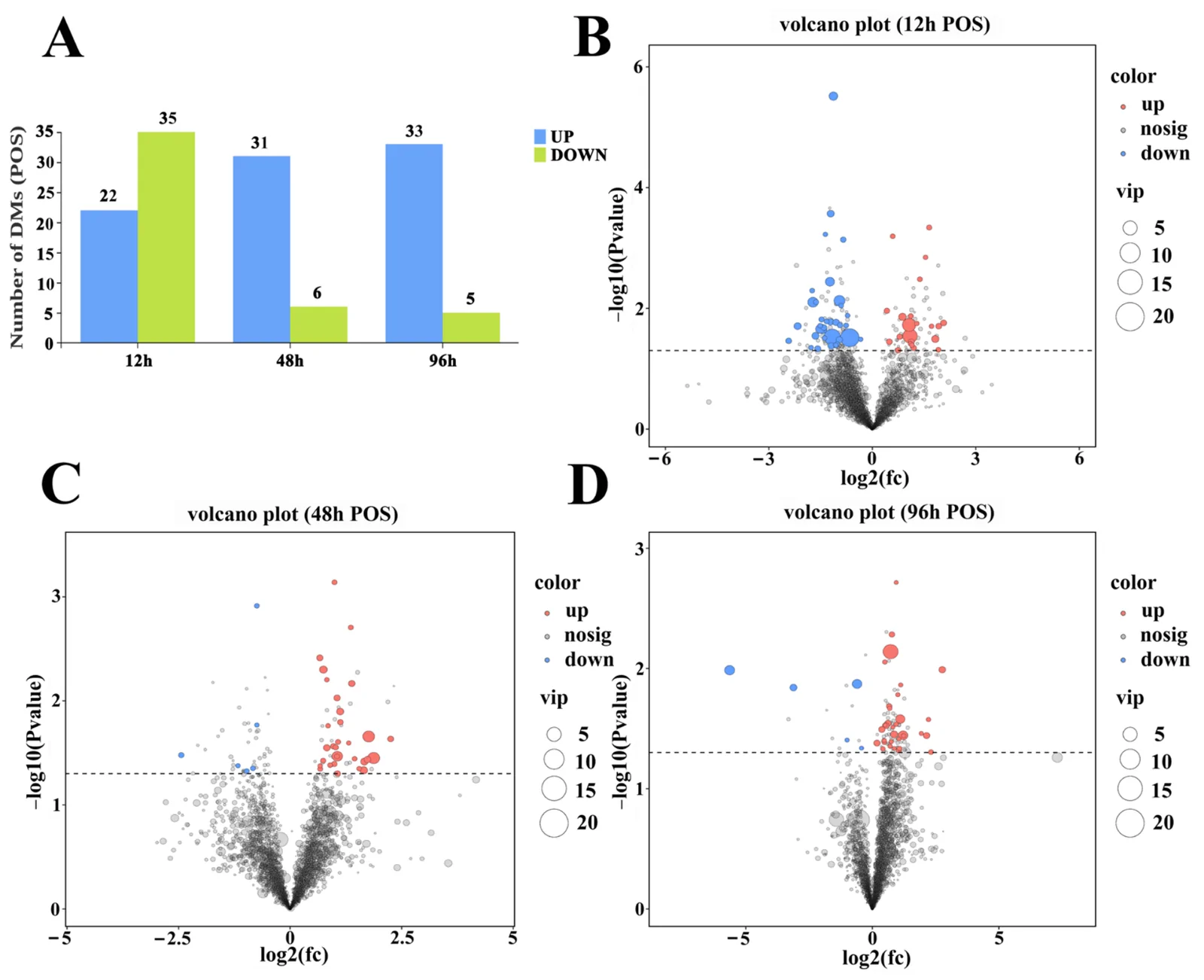
Metabolomic analysis of differential metabolites in POS. (**A**) Up/downregulated metabolite counts at 12, 48, and 96 h. (**B**–**D**) Volcano plots of metabolite distribution (x: log_2_FC; y: −log10(Pvalue)). Colors mark significantly altered metabolites; grey points show non-significant ones.

**Figure 7:**
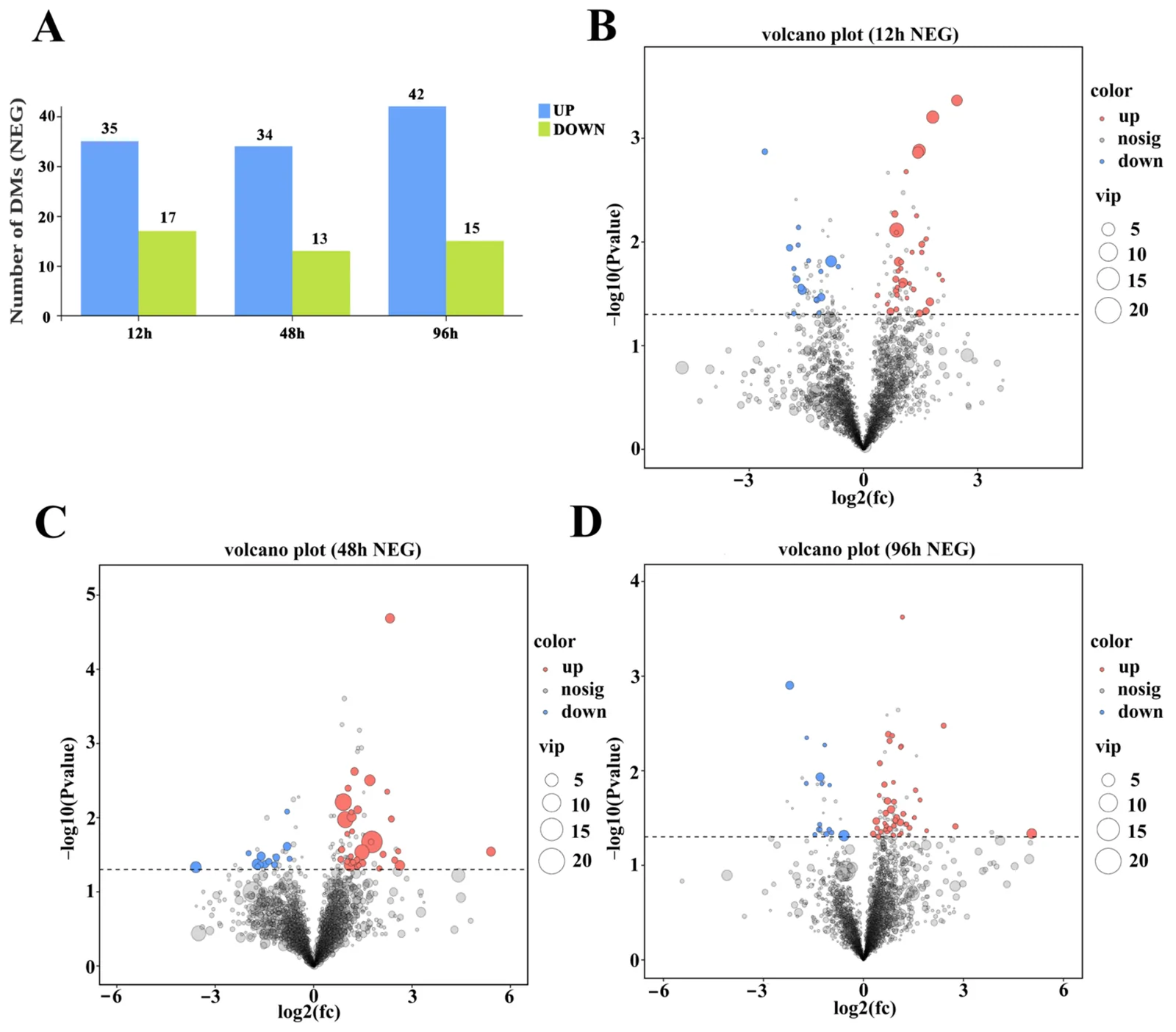
Metabolomics cluster analysis of DAMs in NEG. (**A**) Statistics of upregulated (blue) and downregulated (green) DAMs at 12 h, 48 h, and 96 h compared with the control group. (**B**–**D**) Volcano plots illustrating the overall distribution of DAMs at the three stress time points. The *x*-axis represents log_2_(fold change), and the *y*-axis indicates −log10(Pvalue). Points distributed at both ends of the *x*-axis denote metabolites with larger expression differences. Significantly upregulated and downregulated metabolites are marked with corresponding colors, while gray dots represent non-differential metabolites.

The statistical histogram of differential metabolites revealed that in positive ion mode, 22, 31 and 33 metabolites were significantly up-regulated at 12 h, 48 h and 96 h respectively. Correspondingly, the numbers of markedly downregulated metabolites were 35, 6 and 5 at the above time points (Figure 6A). For negative ion mode, the counts of upregulated DAMs were 35, 34, 42 and downregulated DAMs were 17, 13, 15 (Figure 7A).

Volcano plot analysis in positive ion mode (Figure 6B–D) revealed a temporal pattern of DAMs with an initial downregulation followed by upregulation, showing a clear increasing trend from 48 h onward. In negative ion mode (Figure 7B–D), the overall abundance of DAMs exhibited a continuous increase.

Considering that metabolites differ greatly in ionization properties, many compounds can only be effectively identified under either positive or negative ion mode. Therefore, the metabolic profiles obtained from positive and negative ion modes are displayed separately (Figure 6 and Figure 7) to intuitively reflect the overall metabolite coverage of LC-MS analysis.

### 3.10. KEGG Pathway Analysis of Differential Metabolites

To obtain functional metabolic pathways, we screened DAMs and assigned qualified metabolites to the KEGG pathway database. At 24 h post stress, the predominant DAMs belonged to lipids and lipid-like molecules, purine and adenosine metabolites, and amino acids. Deoxyinosine and several amino acid derivatives (e.g., Glu-Phe, His-Cys, Glu-Gly-Glu) had VIP > 5, representing highly significant DAMs at this time point (Figures S6A and S7A). At 48 h post stress, DAMs mainly belonged to organic heterocyclic compounds, lipids, lipid-like molecules and organic oxygen compounds. Biliverdin, nicotinate, isopyrazam and Ltc4-[d5] showed high VIP values and were identified as key DAMs (Figures S6B and S7B). At 96 h post stress, DAMs were primarily lipid-like molecules, organic acids and derivatives, and amino acids. Glycerophosphocholine, palmitoyl-L-carnitine and Arg-Ser had VIP > 5 and were considered the pivotal differential metabolites at this stage (Figures S6C and S7C).

**Figure 8:**
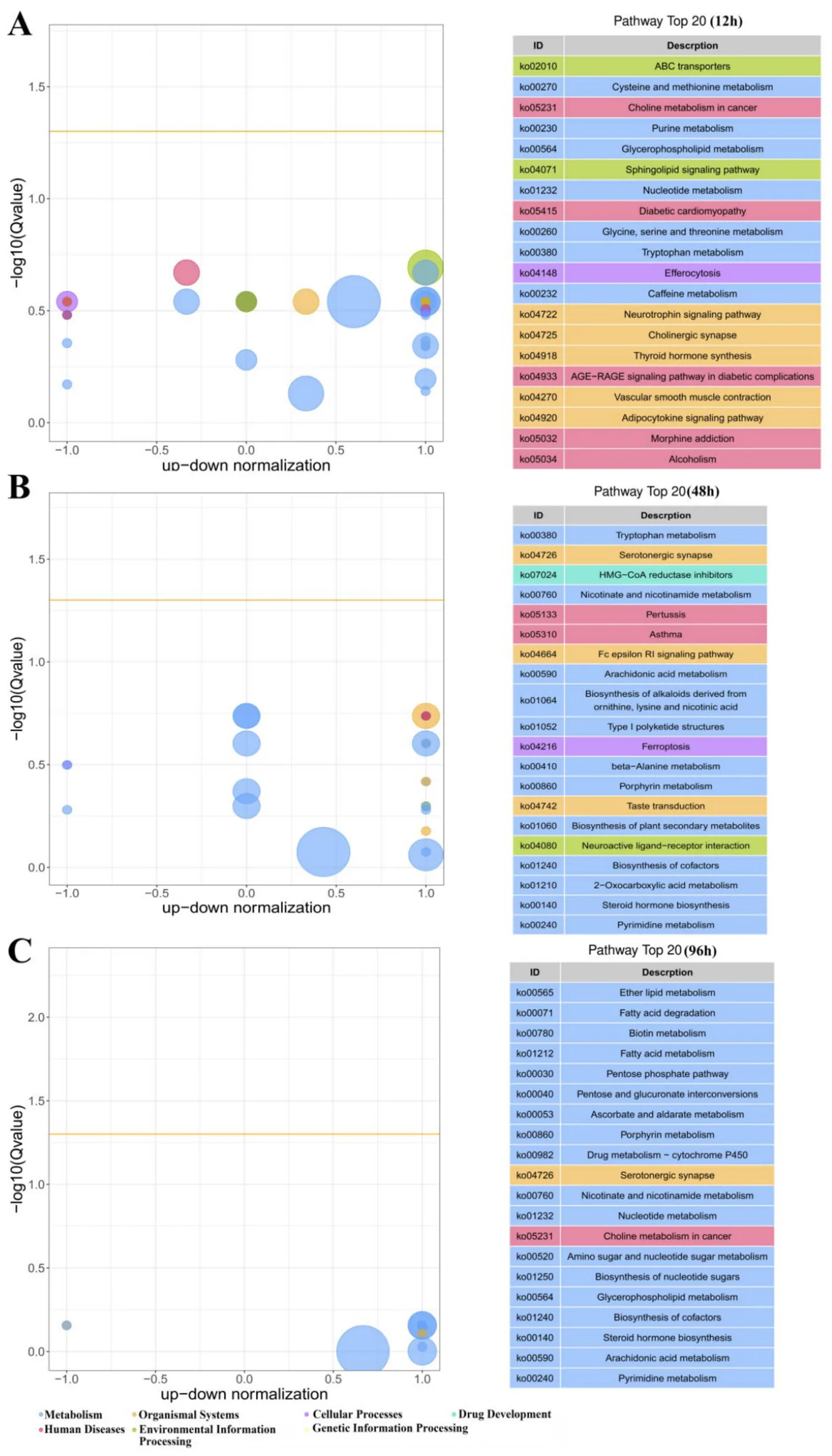
KEGG pathway enrichment bubble plot. (**A**–**C**) KEGG enrichment profiles of metabolites at 12 h, 48 h, and 96 h after low-salinity adaptation and high ammonia nitrogen stress. Pathways with *p* < 0.05 were screened, and the top 20 pathways with the lowest Q-values are presented. The *x*-axis represents the up-down normalization value (i.e., the ratio of the difference between the number of upregulated and downregulated differential metabolites to the total number of differential metabolites), indicating the overall trend of gene expression within the pathway. A positive *x*-axis value indicates a predominance of upregulated genes in the pathway, a negative value indicates a predominance of downregulated genes, and a value close to zero indicates a relatively balanced number of upregulated and downregulated genes. The *y*-axis displays -log10 (Q-value). Higher values correspond to smaller Q-values, indicating stronger enrichment. The yellow line represents the Q-value threshold of 0.05, signifying the statistical significance of pathway enrichment. Bubble size is generally proportional to the number of significantly differentially expressed genes in the pathway; larger bubbles indicate more genes undergoing significant regulation. Bubble color categorizes pathways. The right panel displays the top 20 pathways by Q-value.

In the KEGG enrichment analysis (top 20 pathways, *p* < 0.05), according to the combined KEGG enrichment bubble plot for hepatopancreatic tissues, the markedly altered metabolic pathways under high ammonia stress post low-salinity acclimation were primarily: basal metabolic pathways (especially purine metabolism), ABC transporter pathways, cell membrane maintenance pathways, signal transduction pathways (e.g., glycerophospholipid metabolism), and immune-related pathways (e.g., leishmaniasis) (Figure 8).

### 3.11. Integrated Analysis of Transcriptomic and Metabolomic

This study integrated temporal dynamic changes of dual omics datasets to investigate the internal relationships between genes and metabolites, and characterize putative co-regulatory modules in the hepatopancreas of *L. vannamei* under combined low-salinity acclimation and high ammonia nitrogen stress.

**Figure 9:**
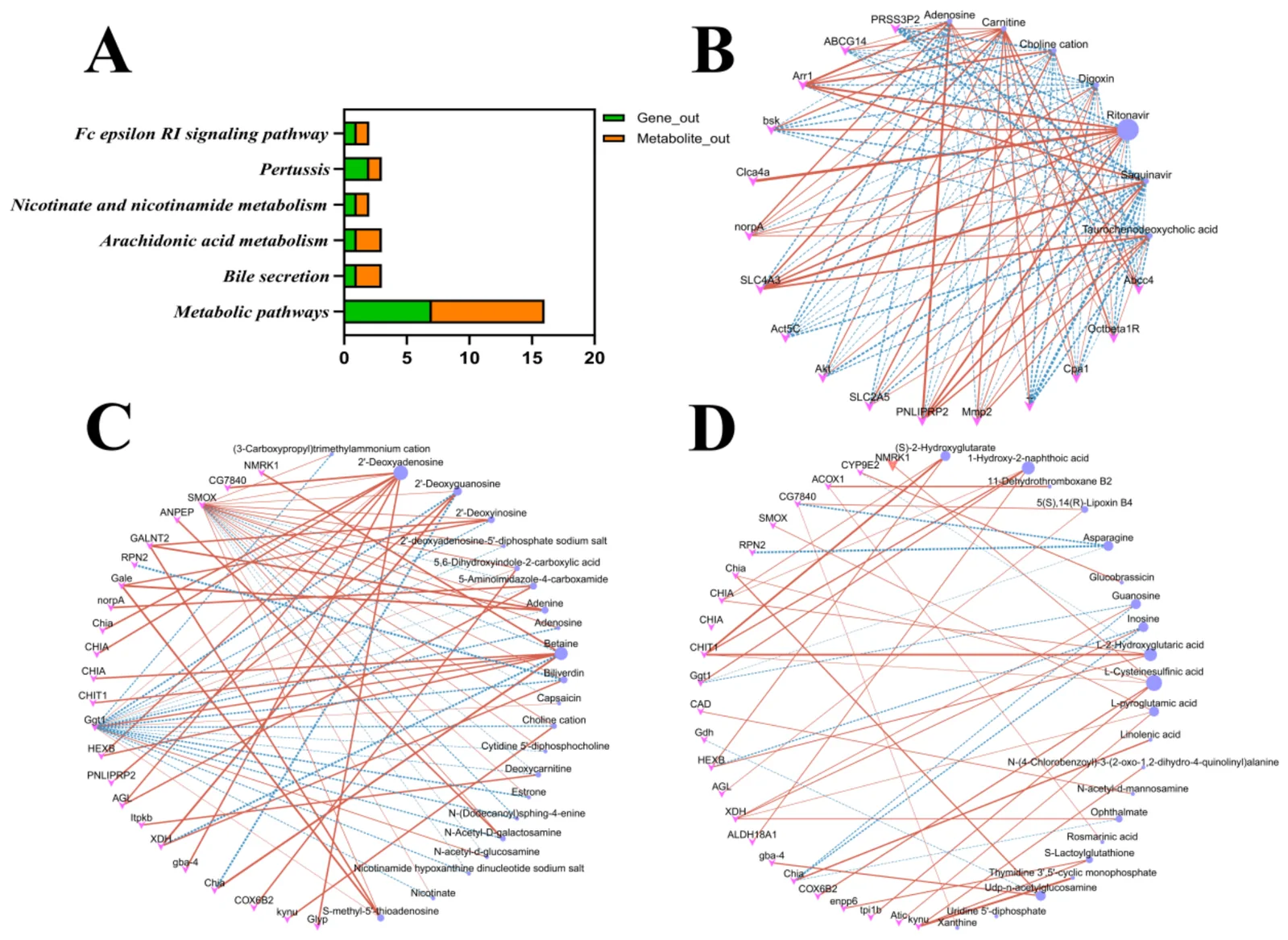
Integrated transcriptome-metabolome analysis for key pathway enrichment and correlation network. (**A**) KEGG pathway enrichment statistics of integrated omics data. Bar length indicates the enrichment number of differentially expressed genes (DEGs, green) and differentially accumulated metabolites (DAMs, orange) in each pathway. The *x*-axis represents the enrichment count of DEGs and DAMs, and the *y*-axis shows the corresponding pathway names. Gene_out and Metabolite_out denote the enriched gene and metabolite numbers within each pathway, respectively. (**B**–**D**) Gene-metabolite correlation networks for screening key DEGs and DAMs. DEGs (|log_2_FC| ≥ 1, *p* < 0.05) and DAMs (VIP ≥ 1, *p* < 0.05) from core pathways were used for network construction, and the top 50 pairs with the highest Pearson correlation coefficients are displayed. In the network, pink arrows represent genes, and blue circles represent metabolites; larger circles indicate higher metabolite connectivity. Red solid lines and blue dashed lines stand for positive and negative correlations, respectively, with line thickness proportional to the correlation strength.

The correlation heatmap between gene expression and metabolite abundance across all samples showed clear clustering, indicating an intrinsic association (Figure S8). Based on the KEGG results from metabolomics and transcriptomics, secretion pathways, metabolic pathways, and immune-related pathways were selected as core target pathways (Figure 9A). Correlation network analysis was performed to reveal the interactions between genes and metabolites. Combined with the Pearson correlation coefficients and *p* values of DEGs and DAMs, the key DEGs and DAMs were further screened (Figure 9B–D). In the secretion pathway, the pivotal DEGs included *slc4a3*, matrix metalloproteinase-17 (*mmp2*), and carboxypeptidase A1 (*cpa1*), with their corresponding key DAMs being saquinavir and carnitine. Among these, *slc4a3* served as the most critical regulatory gene, and the pathway showed a strong positive correlation in the positive ion detection mode, while no significant correlation was observed in the negative ion mode. In the metabolic pathways, five core DEGs were identified. *nmrk1* was positively correlated with betaine and L-cysteinesulfinic acid, but negatively correlated with guanosine. *ggt1* was positively correlated with 1-hydroxy-2-naphthoic acid, and negatively correlated with 2′-deoxyguanosine and asparagine. *gale*, *chia*, and *smox* were all significantly positively correlated with corresponding DAMs. Detailed information is presented in Table 2.

In the immune-related pathways, joint analysis identified the Fc epsilon RI signaling pathway and pertussis as the main immune-associated pathways. The key DEGs screened were *bsk* and *cd29*, with their corresponding DAMs being nicotinate, leukotriene E4, and Ltc4-[d5] (Table 2).

## 4. Discussion

To elucidate the biological mechanisms of *L. vannamei* under high ammonia nitrogen stress after low-salinity acclimation and provide references for future stress-tolerant breeding, this study selected the hepatopancreas as the research subject due to its detoxification, immune, and metabolic functions. We discuss the results from three perspectives: tissue damage and repair, ammonia excretion and detoxification, and antioxidant and immune responses. Integrating physiological and biochemical indicators with transcriptomic and metabolomic data reveals that the hepatopancreas does not counter ammonia toxicity via a single pathway. Instead, it coordinates ion transport, immune regulation, and damage repair into a dynamic “detoxification-defense-repair” network.

### 4.1. Involvement of mmp2 and chia in Tissue Repair and Metabolic Interaction

Ammonia nitrogen stress directly damages the integrity of the basal membrane of hepatopancreatic tubular epithelium. *mmp2* participates in tissue damage repair by regulating extracellular matrix degradation. The role of *mmp* genes in tissue repair has been well documented in zebrafish, tilapia, and crabs [33,34,35]. The coordinated changes between metabolites (e.g., lipid metabolism-related products) and gene expression in the secretion pathway reveal a dynamic coupling between gene transcription and metabolism during hepatopancreatic damage repair.

From the perspective of amino acid and energy metabolism, previous studies have confirmed that ammonia stress impairs intestinal barrier function, and *chia* plays an important role in ammonia toxicity stress responses and intestinal health maintenance [25,36]. In this study, significant differential expression of *chia* was also detected in the hepatopancreas. Moreover, *chia* expression was closely correlated with energy metabolism-related metabolites, including 2′-deoxyadenosine and L-2-hydroxyglutaric acid. This suggests that *chia* may participate in the crosstalk between chitin metabolism and energy metabolism. We hypothesize that the altered *chia* expression in the hepatopancreas under ammonia stress may help maintain metabolic homeostasis and physiological health through the gut-hepatopancreas axis [37].

### 4.2. slc4a3 and ggt1 Modulate Ammonia Metabolism and Detoxification

When crustaceans are exposed to ammonia stress, environmental NH_3_ diffuses across the gill epithelium into the hemolymph along its concentration gradient, elevating hemolymph ammonia levels [32]. Previous studies have shown that hemolymph ammonia content in *L. vannamei* increases significantly with higher ammonia concentrations and prolonged exposure [38]. The trend observed in this study is consistent with previous reports, but the accumulation was more severe. We hypothesize that this difference may be attributed to low-salinity acclimation. Low-salinity conditions impose a greater osmoregulatory burden on *L. vannamei*, which is likely to promote endogenous ammonia accumulation in the hemolymph, as reported in previous studies [39,40]. Notably, owing to the lack of a high-salinity control group in our experimental setup, this inference cannot be directly verified by the present dataset.

The secretion pathway plays a key role in ammonia excretion. *slc4a3*, encoding a bicarbonate transporter, is the most critical gene in this pathway and reflects ion regulation in the hepatopancreas under ammonia stress [41]. It is inferred that ammonia stress induces intracellular acid-base imbalance, which drives *slc4a3* upregulation to maintain cellular pH homeostasis. Carnitine, the key metabolite corresponding to this gene and an essential carrier for fatty acid β-oxidation, accumulated simultaneously. This likely represents a compensatory response in the hepatopancreas that provides substrates for energy metabolism, thereby supporting the energy-demanding processes of ammonia excretion and detoxification [17,42].

Crustaceans also detoxify ammonia directly by converting it into low-toxicity metabolites such as glutamine, urea, and uric acid [14]. In this study, hemolymph urea nitrogen and uric acid levels gradually increased with prolonged stress, indicating that *L. vannamei* activated both urea- and uric acid-based detoxification pathways. Previous studies have reported that under ammonia stress, hemolymph urea content increases with rising ammonia concentrations in *L. vannamei* and *Macrobrachium nipponense*, while glutamine synthesis in the hepatopancreas is significantly inhibited [43,44], consistent with our findings.

γ-GT is widely involved in glutathione metabolism, amino acid transport, and indirect ammonia detoxification, serving as an important physiological indicator of detoxification capacity in aquatic organisms [31]. Studies have confirmed that γ-GT activity effectively evaluates environmental stress tolerance and detoxification potential in crustaceans and fish. For example, γ-GT has been used as a toxicity response biomarker in *Eriocheir sinensis* [31] and *L. vannamei* [45]. Similarly, in ammonia stress research, γ-GT is commonly used to assess metabolic adaptation and detoxification levels in *Oreochromis niloticus* [46], *Megalobrama amblycephala* [41], and *Seriola dumerili* [47]. *ggt1* is the key structural gene encoding this enzyme. In this study, γ-GT activity peaked at 12 h of stress exposure, indicating that early ammonia stress rapidly activated γ-GT via post-translational modification to enhance the γ-glutamyl cycle, thereby strengthening ammonia detoxification in the hepatopancreas. However, *ggt1* expression was slightly downregulated at this stage, which may be attributed to transient feedback regulation of the γ-glutamyl cycle under acute ammonia stress. With prolonged stress, continuous ammonia exposure suppressed *ggt1* transcription and enzyme synthesis, leading to a gradual decline in γ-GT activity and reduced detoxification capacity [48]. Notably, the subsequent recovery of *ggt1* expression suggests that *L. vannamei* may reestablish physiological homeostasis through remodeling of glutathione and nitrogen metabolism.

### 4.3. Influence of Oxidation and JNK-Integrin

As a key intracellular non-enzymatic antioxidant in hemolymph, GSH has dual functions: directly scavenging ROS and providing substrates for glutathione S-transferase (GST) and glutathione peroxidase (GSH-Px) [49]. In this study, hemolymph GSH content continuously decreased with prolonged stress, indicating that the antioxidant defense system of *L. vannamei* was severely challenged. In contrast, the lipid peroxidation marker MDA content gradually increased over time. This trend is consistent with previous reports on *L. vannamei* and *Macrobrachium rosenbergii* under ammonia stress [13,50,51].

Ammonia nitrogen stress disrupts the mucosal barrier of the hepatopancreas in *L. vannamei* and causes structural damage [52,53]. Studies have shown that ammonia stress can trigger activation of the c-Jun N-terminal kinase (JNK) signaling pathway in tissues such as the intestine, thereby inducing apoptosis and inflammatory responses [54]. In the present study, transcriptional regulation of JNK-related gene *bsk* was observed in hepatopancreas, although *bsk* was down-regulated at 48 h. It should be noted that alterations in *bsk* mRNA abundance do not directly reflect functional activation of the JNK cascade, as JNK activation is primarily driven by protein phosphorylation. Although the direct function of *bsk* homologs in crustaceans has not been identified, the hepatopancreas of *L. vannamei* also exhibits typical apoptotic features under ammonia stress, suggesting that the JNK signaling cascade may exert a potential regulatory role [53,55]. Furthermore, integrin β (e.g., CD29/integrin β1), as a key cell adhesion molecule, participates in hemocyte adhesion and immune signal transduction in shrimp [56].

### 4.4. Additional General Features of Hepatopancreatic Adaptive Responses

Beyond the core regulatory network described above, two notable patterns in our data deserve further clarification. First, the activities of three key ammonia-metabolizing enzymes (GDH, GS, and γ-GT) all show a trend of initial increase followed by decrease. From a biological perspective, this may reflect a two-phase adaptive strategy in response to ammonia stress following adaptation to low salinity: in the early stages of stress, existing enzyme proteins are rapidly activated through post-translational regulation to facilitate ammonia detoxification and counteract acute ammonia toxicity. As ammonia stress persists, although the osmotic regulatory system has stabilized following adaptation to low salinity, continued ammonia exposure still results in sustained metabolic expenditure. The hepatopancreas thus undergoes active metabolic remodeling, gradually downregulating the energy-intensive glutamine synthesis pathway and shifting to relatively lower-cost detoxification routes such as urea and uric acid, which matches the subsequent decline in enzyme activities [21,42].

Second, at the 48 h, the number of downregulated genes far exceeded the number of upregulated genes. This phenomenon may suggest that the hepatopancreas has passed through the acute stress response phase. Rather than continuing to significantly activate genes, it conserves energy by suppressing non-essential functions such as growth and biosynthesis, maintaining activity only in core detoxification and repair genes [10,53].

### 4.5. Strengths, Limitations, and Future Work

This work elucidates the molecular mechanisms of *L. vannamei* exposed to high ammonia stress after low-salinity acclimation, and identifies core genes and metabolites associated with stress tolerance. These indicators can assess shrimp physiological status and serve as candidate biomarkers for breeding stress-tolerant strains. The findings also provide theoretical guidance for ammonia control and feeding management in low-salinity aquaculture.

Several limitations exist. First, only the hepatopancreas was analyzed; other tissues (e.g., intestine) may differ. Second, individual shrimp variation may introduce minor bias despite randomization. Third, functional annotations rely on homology predictions from model organisms, requiring cautious interpretation. Notably, metabolite annotations based solely on untargeted LC-MS/MS spectral matching are tentative. For example, the feature tentatively annotated as saquinavir should not be considered definitive identification. Correlations between genes and ambiguously annotated metabolites require cautious interpretation and cannot be directly inferred as physiological interactions in shrimp. Fourth, molecular mechanisms need further validation (e.g., gene knockdown). Additionally, the use of 96 h LC_50_ simulates acute ammonia spikes; responses under chronic exposure may differ. Though existing limitations, the main findings remain valid and can direct further research.

In future studies, we will perform gene knockdown experiments targeting core candidate genes including *ggt1*, to further verify their functional roles in ammonia detoxification and immune regulation in *L. vannamei*.

## 5. Conclusions

After adapting to low salinity, *L. vannamei* were challenged with high ammonia nitrogen, and their physiological and molecular responses were analyzed in this research. Exposure to high ammonia nitrogen disrupted hemolymph biochemistry and modulated enzyme activities related to antioxidation and metabolism, which impaired hepatopancreatic functions. Integrated multi-omics analysis uncovered three core pathways related to secretion, basal metabolism and immunity, along with 11 pivotal genes (e.g., *slc4a3*, *ggt1*, *chia*, *bsk*) and 17 markedly differential metabolites (e.g., 2′-deoxyguanosine, L-pyroglutamic acid). These genes and metabolites collectively regulate the responses of shrimp to ammonia nitrogen stress in tissue repair, antioxidant defense and metabolic processes. It further reveals the molecular mechanisms of ammonia stress response in low-salinity-acclimated *L. vannamei*. It provides a theoretical basis for the eco-friendly and sustainable culture of shrimp in estuarine regions, and offers practical guidance for water quality maintenance and ammonia nitrogen management in aquaculture. In practical low-salinity estuarine farming, special attention should be paid to strict ammonia nitrogen control during the middle-late culture stage; even relatively low environmental ammonia concentrations may produce severe toxic effects on low-salinity-acclimated shrimp, so as to alleviate stress-related mortality and improve economic efficiency.

## Figures and Tables

**Table 1:** Representative genes with significant differential expression.

			log_2_ratio		
Accession Number	Gene Name	Description	12 h	48 h	96 h
Secretion					
ncbi_113813287	*slc* *4* *a* *3*	SLC4A2, AE2; solute carrier family 4 (anion exchanger), member 2	−1.33	−0.79	0.28
ncbi_113805827	*c* *pa1*	CPA1; carboxypeptidase A1	0.78	−1.63	−0.92
ncbi_113808646	*m* *mp2*	MMP17; matrix metalloproteinase-17 (membrane-inserted)	0.14	−2.48	3.83
Metabolic pathways					
ncbi_113825972	*smox*	SMOX, PAO5; spermine oxidase	4.97	−2.53	−0.19
ncbi_113817258	*chia*	E3.2.1.14; chitinase	0.64	−1.86	−0.56
ncbi_113817475	*g* *ale*	galE, GALE; UDP-glucose 4-epimerase	−1.10	−4.45	1.01
ncbi_113865456	*nmrk* *1*	NRK1_2; nicotinamide/nicotinate riboside kinase	0.62	−2.25	−0.31
ncbi_113815919	*ggt1*	GGT1_5, CD224; gamma-glutamyltranspeptidase/glutathione hydrolase /leukotriene-C4 hydrolase	−0.54	−2.12	0.26
ncbi_113861480	*ada* *2*	CECR1, ADA2; adenosine deaminase CECR1	−0.19	−1.55	0.41
ncbi_113815222	*gbd*	E1.14.11.1; gamma-butyrobetaine dioxygenase	1.74	4.02	−0.77
ncbi_113804456	*tpmt*	TPMT, tpmT; thiopurine S-methyltransferase	−1.57	−0.35	−0.87
Protein digestion and absorption					
ncbi_113815728	*let-2*	COL4A; collagen, type IV, alpha	−0.40	−2.59	2.75
ncbi_113813021	*a* *ct5* *c*	ACTB_G1; actin beta/gamma 1	0.04	1.21	−0.46
ncbi_113827072	*c* *ol5a1*	COL5A5; collagen, type V/XI/XIV/XXVII, alpha	0.11	−3.46	2.77
ncbi_113818142	*col* *4* *a*	COL4A; collagen, type IV, alpha	−0.13	−3.61	1.70
ncbi_113807987	*col2a1*	COL2A; collagen, type II, alpha	0.06	−4.62	3.02
Mismatch repair					
ncbi_113813628	*rcf* *1*	RFC1; replication factor C subunit 1	−0.03	−2.15	13.37
Immune limited gene					
ncbi_113807721	*rps* *6* *ka* *3*	RPS6KA; ribosomal protein S6 kinase alpha-1/2/3/6	0.27	−1.01	−0.06
ncbi_113814567	*cd* *29*	ITGB1, CD29; integrin beta 1	−2.31	−5.94	−1.96
ncbi_113819710	*bsk*	JNK; mitogen-activated protein kinase 8/9/10 (c-Jun N-terminal kinase)	0.54	−1.84	−0.20
ncbi_113814443	*l* *an* *b* *2*	LAMC1; laminin, gamma 1	−0.49	−1.53	0.25
ncbi_113802906	*l* *an* *a*	LAMA3_5; laminin, alpha 3/5	−0.32	−2.08	0.95
ncbi_113800896	*w* *ee1*	WEE1; wee1-like protein kinase	0.54	−1.49	−0.21
ncbi_113805114	*insr*	INSRR; insulin receptor-related receptor	−0.68	−1.49	0.08

**Table 2:** Integrated analysis of key DEGs and key DAMs.

Pathway	Key DEGs	Key DAMs	
Correlation		Positive	Negative
secretion	*slc4a3*	Saquinavir	-
	*cpa1*	-	Carnitine
	*clca4a*	Ritonavir	-
	*mmp2*	Carnitine	-
Metabolic pathway	*nmrk1*	Betaine; L-Cysteinesulfinic acid	Guanosine;
	*ggt1*	1-Hydroxy-2-naphthoic acid	2′-Deoxyguanosine; Guanosine; Asparagine
	*gale*	S-methyl-5′-thioadenosine; 2′-Deoxyinosine;Adenine	-
	*chia*	2′-Deoxyadenosine; L-2-Hydroxyglutaric acid; (S)-2-Hydroxyglutarate; 1-Hydroxy-2-naphthoic acid	-
	*smox*	L-pyroglutamic acid	-
Immune pathway	*bsk*	Ltc4-[d5]	Nicotinate;Leukotriene E4
	*cd29*	Nicotinate;Leukotriene E4	Ltc4-[d5]

-: no corresponding metabolites detected.

## Data Availability

The datasets supporting the findings of this work are available from the corresponding author on reasonable request. Transcriptomic data have been deposited in the NCBI Sequence Read Archive (SRA) under BioProject accession [PRJNA1471518]. The data are publicly available at: https://www.ncbi.nlm.nih.gov/bioproject/PRJNA1471518 (accessed on 11 August 2026). Metabolomic data have been deposited in the MetaboLights database under study accession [MTBLS14736]. The data are publicly available at: https://www.ebi.ac.uk/metabolights/MTBLS14736 (accessed on 11 August 2026). The dataset will be released publicly upon publication of this manuscript.

## Supplementary Materials

The following supporting information can be downloaded at: https://www.mdpi.com/article/10.3390/biology15161394/s1, Table S1. Concentrations and cumulative mortality in the preliminary experiment; Table S2. Summary of measured parameters, detection wavelengths, and units; Table S3. Primers used for qRT-PCR; Table S4. RNA-seq sequencing data. Table S5. Metabolite identification results; Figure S1. Transcriptome quality assessment; Figure S2. Distribution of gene expression abundance; Figure S3. GO enrichment analysis of the transcriptome; Figure S4. qRT-PCR validation of representative DEGs; Figure S5. Metabolome Correlation Heatmap; Figure S6. VIP Score Plots of Differentially Expressed Metabolites in POS; Figure S7. Statistical Plot of VIP Values for Differentially Expressed Metabolites in NEG; Figure S8. Heatmap of correlations between differentially expressed genes and metabolites.
