# Supplementary material for: Physiological, Transcriptomic, and Metabolomic Insights into Ammonia-Nitrogen Stress in the Hepatopancreas of Litopenaeus vannamei Acclimated to Low Salinity
Source: Biology (Basel). 2026 Aug 14;15(16):1394. doi: 10.3390/biology15161394 (PMC13509465; doi:10.3390/biology15161394)
Supplement: Supplementary file 1 [file biology-15-01394-s001.zip › biology-4480033-supplementary.pdf]

Table S1. Concentrations and cumulative mortality in the preliminary experiment

TAN concentrations (mg/L)	cumulative mortality rates (%)
0	3.3
5	10.0
10	16.7
15	23.3
20	29.0
30	38.0
40	47.0
50	62.0
60	83.3

Table S2. Summary of measured parameters, detection wavelengths, and units

name	detection wavelength (nm)	Unit
CATase	405nm	U/mgprot
SODase	550nm	U/mgprot
GDHase	340nm	U/mgprot
GSase	540nm	μ mol/h/mgprot
γ-GTase	415nm	U/prot
Protein concetration	595nm	mgprot/ml
MDA	532nm	nmol/ml
GSH	405nm	μ mol/L
Ammonia	630nm	μ mol/L
Urea nitrogen	520nm	mmol/L
Uric acid	546nm	μ mol/L

Table S3. Primers used for qRT-PCR

Primer name	Sequence (5′ to 3′)	Length (bp)
Cpa1-F	CACCTGGCACAATGACAGAC	20
Cpa1-R	CTGAGGTCGGTCGTGAAGTC	20
Mmp2-F	CCTGGAAAGACGGAGTGTCG	20
Mmp2-R	AGTACTCCACGGCGTTCTTG	20
CHIA-F	TACCTGTCCCTCGGTCTTCC	20
CHIA-R	GGAGTCGTTCGCAGATCTCAT	21
Gale-F	GTTGATGGCACAGGTGTACG	20
Gale-R	CCAGCGTTGCTACATCTCCT	20
Ggt1-F	TGAAGAGGAATGGCACAGCA	20
Ggt1-R	GCCCACCATTTTCCTTTAGCTTT	23
let-2-F	CATCAGGATCTGGGTCTGGC	20
let-2-R	GCATCATGGGAATGGGGTCA	20
col2a1-F	CAGAACTCCCAAGTGGCGAA	20
col2a1-R	TGGTTGTTGTCCGCCTTGTA	20
RFC1-F	AGGGGGCCAATGATGTCTAT	20
RFC1-R	CACATCCCATGGACCCATTTT	21
RPS6KA3-F	AAGCGGATGGCGACTACAAT	20
RPS6KA3-R	GTACTCTGTCCCGCACCTTC	20
CD29-F	ACTGGTTGTGTCCTGGTGAC	20
CD29-R	CACACCTGCAAATGACCTGC	20
bsk-F	TATGTGGAAAACCGTCCCCG	20
bsk-R	CGTGCTTGACTTGCTTTGAGT	21
SLC4A3-F	CAAGTGCTTACAGGAGGCCA	20
SLC4A3-R	TTGATCCAACGAGCGGTCTC	20
β-actin-F	GCCCATCTACGAGGGATA	18
β-actin-R	GGTGGTCGTGAAGGTGTAG	19

Table S4. RNA-seq sequencing data.

Sample	RawData (bp)	CleanData (%)	LowQuality (%)	GC(%)	Q20(%)	Q30(%)	Total_Mapped(%)
12 h-CK	6,867,982,470.00	100.00	0.00	48.24	99.41	97.79	88.41
48 h-CK	6,550,022,561.00	100.00	0.00	47.39	99.34	97.54	88.51
96 h-CK	6,842,355,547.00	100.00	0.00	47.31	99.40	97.76	88.74
12 h	6,614,492,530.00	100.00	0.00	47.67	99.47	97.94	89.12
48 h	6,815,957,134.00	100.00	0.00	46.69	99.44	97.94	89.43
96 h	6,886,113,314.00	99.99	0.00	47.70	99.41	97.72	89.66

Table S5. Metabolite identification results

Type	All	Known	Unknown
POS	11802	3341	8461
NEG	12375	3185	9217

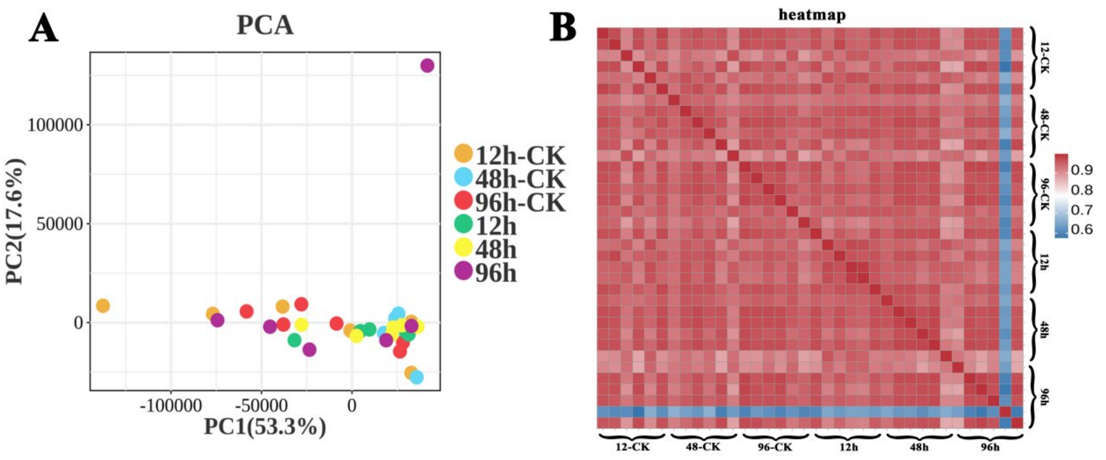

Figure S1. Transcriptome quality assessment. (A) Transcriptome PCA analysis. The Y-axis labels 12 h, 48 h, 96 h represent the experimental group subjected to high ammonia nitrogen stress under low-salinity at three time points. The labels 12 h-CK, 48 h-CK, 96 h-CK represent the control groups held in 5‰ salinity aquaculture water without high ammonia nitrogen stress at the corresponding time points. (B) Transcriptome Correlation Heatmap. The Y-axis labels 12 h, 48 h, and 96 h represent the experimental groups subjected to high ammonia nitrogen stress under low-salinity at corresponding time points. The labels 12 h-CK, 48 h-CK, and 96 h-CK represent the control groups held in 5‰ salinity aquaculture water without high ammonia nitrogen stress at the corresponding time points. The expression levels of each pair of samples were extracted to calculate the Pearson correlation coefficient between every two samples. These coefficients were then visually presented in the form of a heatmap to reflect the correlation between any two samples, which allows for the evaluation of the repeatability of intra-group replicate samples.

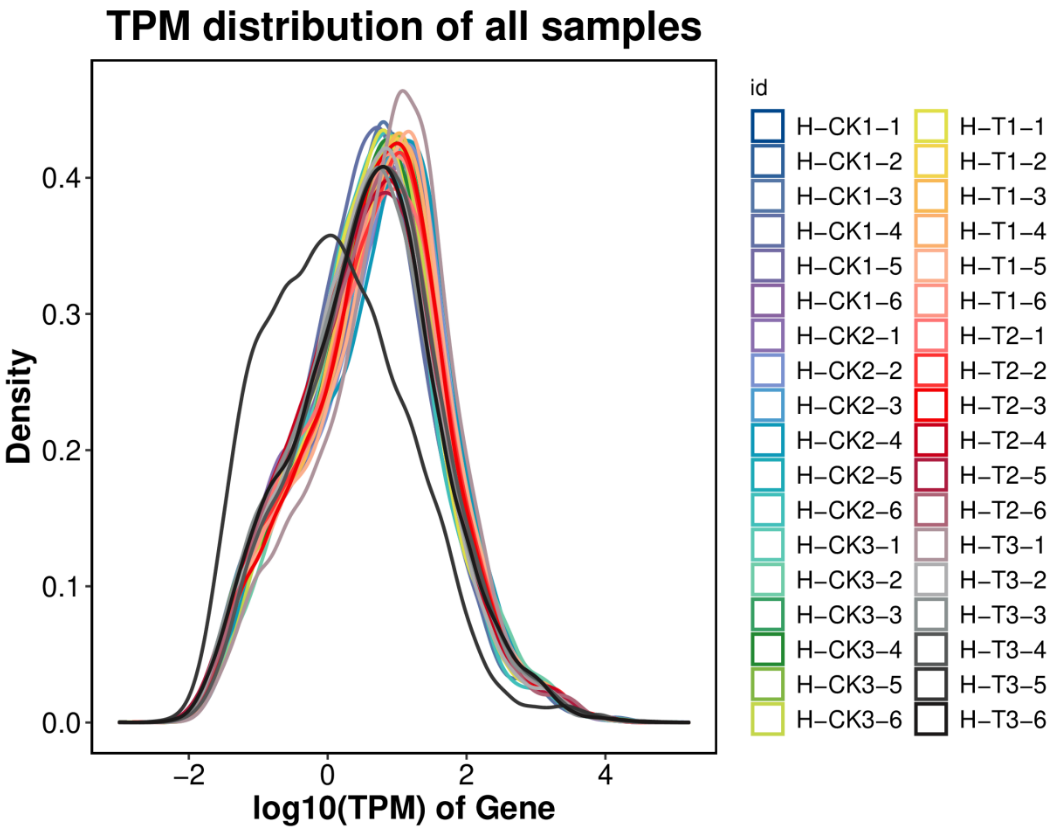

Figure S2. Distribution of gene expression abundance. The X-axis represents log10(TPM), where higher values indicate higher gene expression levels. The Y-axis denotes gene abundance, calculated as the proportion of genes with the corresponding expression level relative to the total number of detected genes. Each colored curve corresponds to an individual sample. In the experimental design, both the control and treatment groups included six biological replicates at each time point. H-CK represents the control group, with CK1, CK2, and CK3 corresponding to the 12 h, 48 h, and 96 h control time points, respectively; H-T represents the treatment group, with T1, T2, and T3 corresponding to the 12 h, 48 h, and 96 h treatment time points, respectively. The peak of each distribution curve indicates the dominant expression range where genes are most concentrated across all samples, reflecting the typical transcriptional abundance level of the dataset.

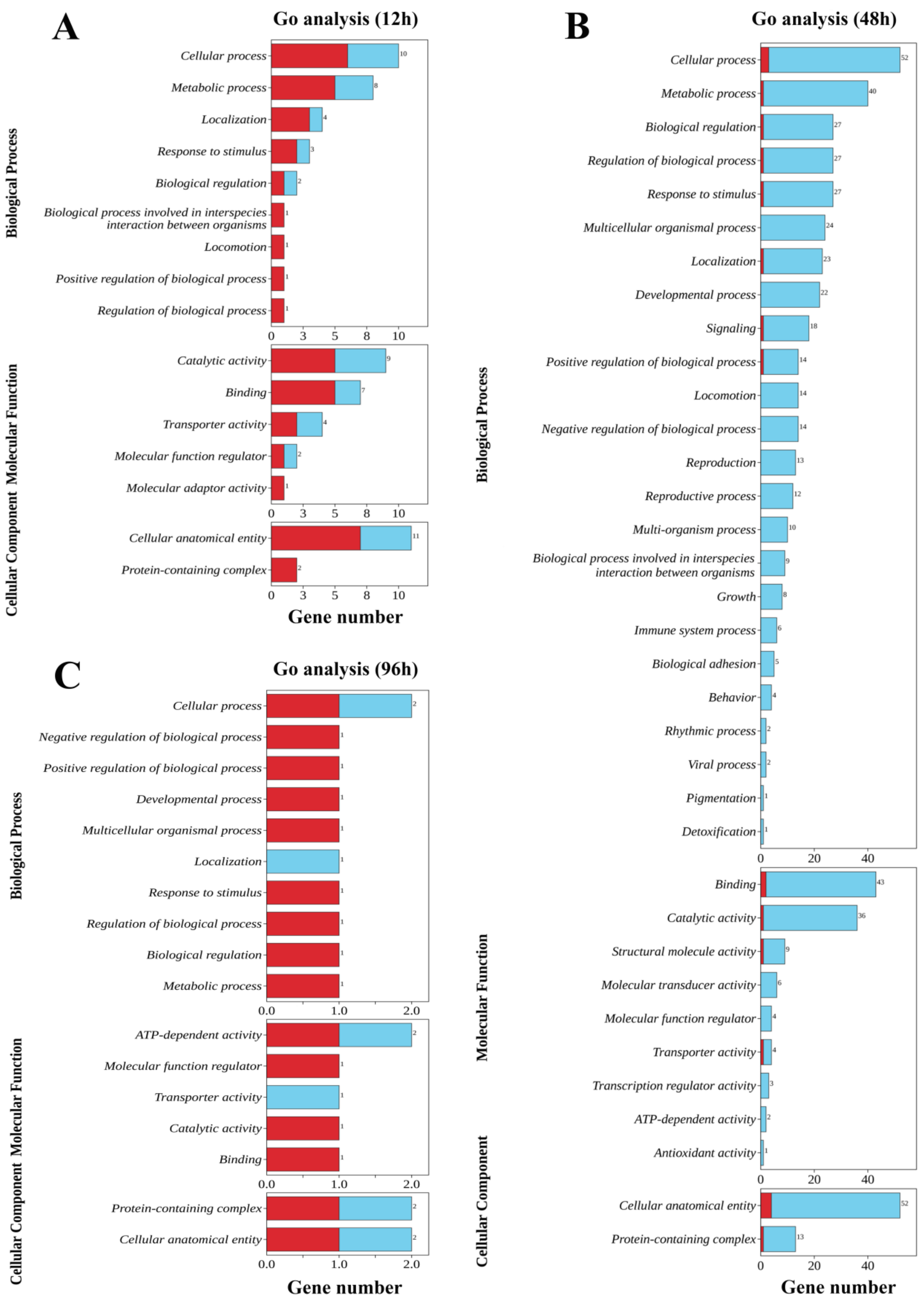

Figure S3. GO enrichment analysis of the transcriptome. (A-C) depict the results of GO enrichment analysis for *L. vannamei* under high ammonia nitrogen stress at 12 h, 48 h, and 96 h under low-salinity conditions. All enrichment results were classified into three core functional ontologies of the GO database: biological process (BP), cellular component (CC) and molecular function (MF). The y-axis of the plots represents the respective subcategories under these three core functional ontologies. In the figures, red bar refers to the number of upregulated genes in the corresponding functional subcategory at the given stress time point, while blue bar

represents the number of downregulated genes in the relevant functional subcategory at the specified stress time point. The numbers displayed on these columns represent the total number of significantly differentially expressed genes within the respective GO analysis subcategory.

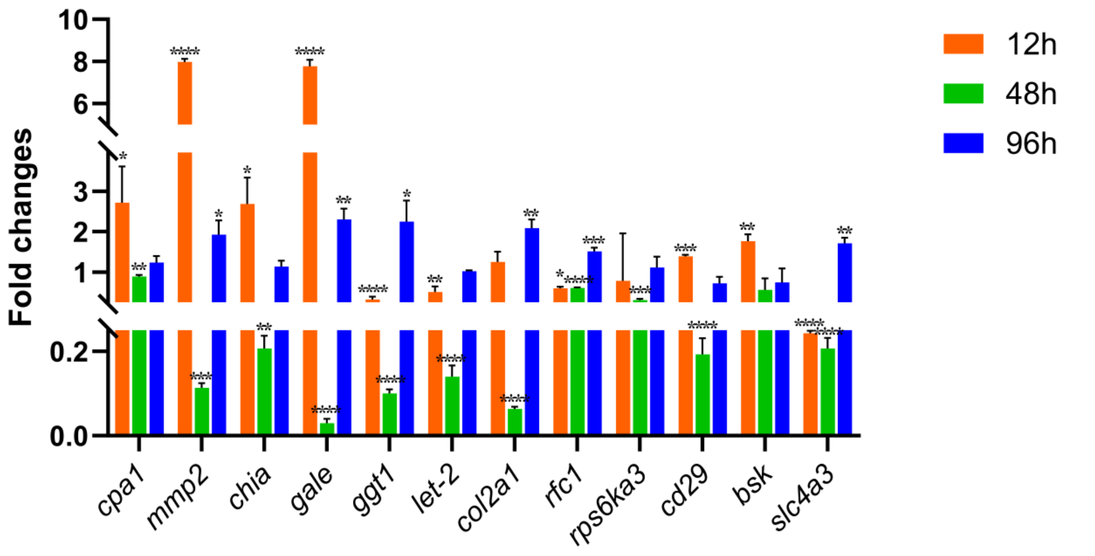

Figure S4. qRT-PCR validation of representative DEGs. The X-axis shows 12 representative DEGs randomly selected from Table 1, which were validated by quantitative real-time PCR (qRT-PCR). The Y-axis represents the fold change, calculated as the relative expression of each gene in the treatment group relative to the control group at each stress time point based on Ct values. In the figure, orange bars represent the fold change results at 12 h post-stress, green bars at 48 h post-stress, and blue bars at 96 h post-stress. Statistical significance was assessed via a two-tailed *t*-test for each stress time point by comparing the treatment group to the control group. Significance levels are annotated as follows: $p < 0.0001$ denoted by "****"; $p < 0.001$ denoted by "***"; $p < 0.01$ denoted by "**"; $p < 0.05$ denoted by "*".

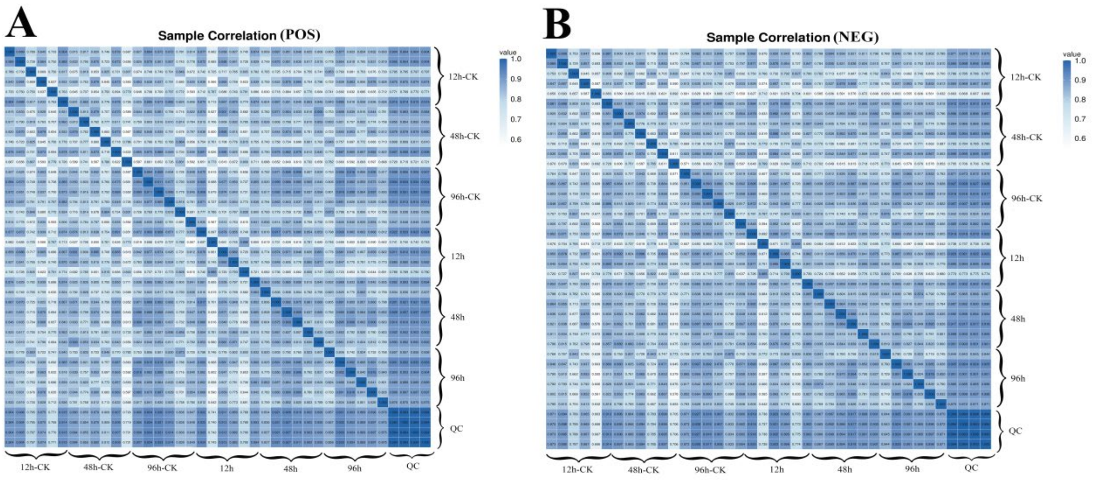

Figure S5. Metabolome Correlation Heatmap. (A) shows the correlation heatmap for the positive ion mode (POS); (B) shows the correlation heatmap for the negative ion mode (NEG). The Y-axis labels 12 h, 48 h, and 96 h represent the experimental group subjected to high

ammonia nitrogen stress under low salinity at three time points. The labels 12 h-CK, 48 h-CK, and 96 h-CK denote the control group maintained only in 5‰ salinity aquaculture water without high ammonia nitrogen stress at the same three time points. The expression levels of each pair of samples were extracted to calculate the Pearson correlation coefficient between every two samples. These coefficients were then visually presented in the form of a heatmap to reflect the correlation between any two samples, which allows for the evaluation of the repeatability of intra-group replicate samples.

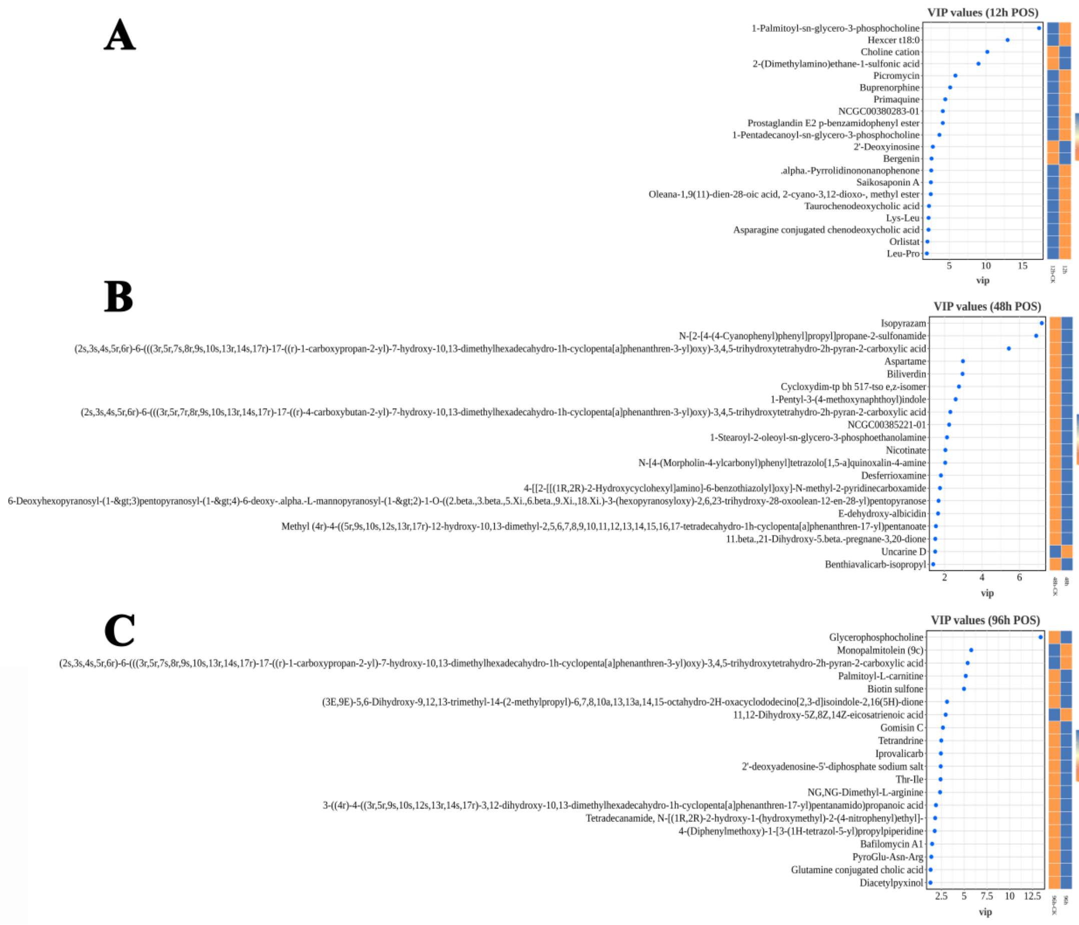

Figure S6. VIP Score Plots of Differentially Expressed Metabolites in POS. (A) VIP score plot of DEMs in positive ion mode at 12 h post-ammonia nitrogen stress; (B) at 48 h post-ammonia nitrogen stress; (C) at 96 h post-ammonia nitrogen stress.ModeOrthogonal partial least squares discriminant analysis (OPLS-DA) was performed to generate the Variable Importance in Projection (VIP) score for each metabolite. Higher VIP scores reflect a stronger contribution of metabolites to the discrimination between the control and treatment groups. Metabolites with a VIP > 1 were defined as key differentially expressed metabolites (DMs), as they exhibit substantial discriminatory power for sample grouping.VIP score plots visually quantify the relative importance of DMs and their individual contributions to sample differentiation. The X-axis represents VIP scores, while the Y-axis lists the top 20 DMs ranked by their VIP scores. Metabolic abundances were first normalized by calculating the group mean (average of all biological replicates within each group) and subsequently subjected to z-score transformation for

colorimetric visualization. Color coding on the right side denotes the relative abundance changes of metabolites: blueindicates upregulation, and orange indicates downregulation.

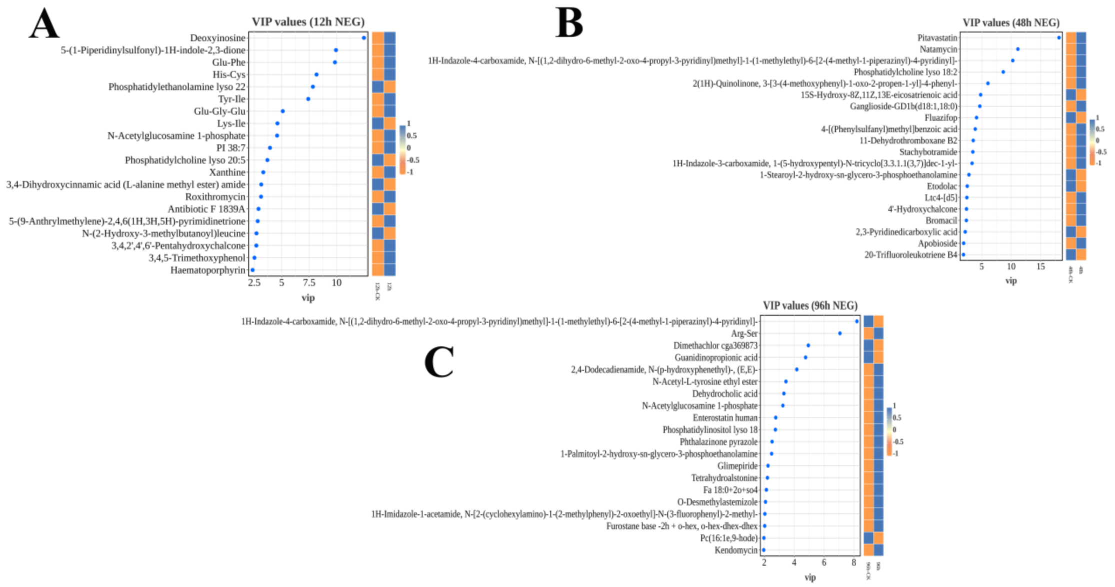

Figure S7. Statistical Plot of VIP Values for Differentially Expressed Metabolites in NEG. (A) VIP plot of differentially expressed metabolites under negative ion mode at 12 h post-stress; (B) VIP plot of differentially expressed metabolites under negative ion mode at 48 h post-stress; (C) VIP plot of differentially expressed metabolites under negative ion mode at 96 h post-stress.Through OPLS-DA analysis, each metabolite yields a VIP value (Variable Importance in Projection). A higher VIP value indicates greater contribution to distinguishing between groups. Metabolites with VIP > 1 exhibit significant differences. The VIP plot illustrates the importance of differentially expressed metabolites and their contribution to sample differentiation. The X-axis displays VIP values, while the Y-axis lists the top 20 differentially expressed metabolites. Metabolic abundance was averaged across samples within each group, followed by z-score analysis. Colors on the right indicate relative abundance changes: blue denotes upregulation, orange indicates downregulation.

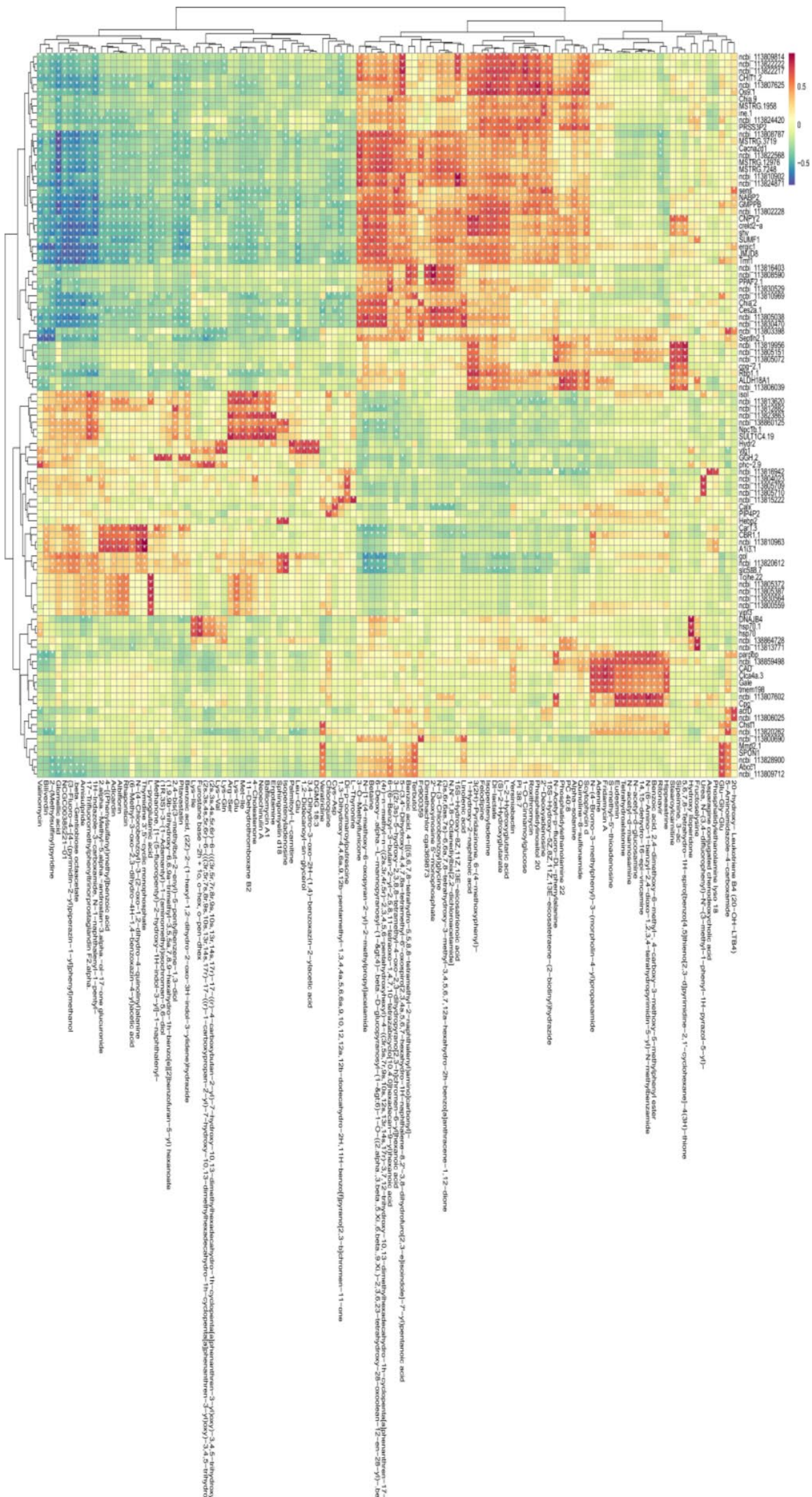

Figure S8. Heatmap of correlations between differentially expressed genes and metabolites. The top 250 differentially expressed genes and metabolites ranked by correlation coefficient are visualized as a heatmap; the x-axis displays metabolite names, while the y-axis shows NCBI IDs of differentially expressed genes. The gradient colors indicate the strength of the correlation between genes and metabolites; the redder the color, the stronger the positive correlation, and the bluer the color, the stronger the negative correlation.Correlation p-values are displayed using “*” symbols. A higher number of “*” indicates a more significant correlation between the gene and metabolite.
